# Synaptotagmin-11 is a critical mediator of parkin-linked neurotoxicity and Parkinson’s disease-like pathology

**DOI:** 10.1038/s41467-017-02593-y

**Published:** 2018-01-08

**Authors:** Changhe Wang, Xinjiang Kang, Li Zhou, Zuying Chai, Qihui Wu, Rong Huang, Huadong Xu, Meiqin Hu, Xiaoxuan Sun, Suhua Sun, Jie Li, Ruiying Jiao, Panli Zuo, Lianghong Zheng, Zhenyu Yue, Zhuan Zhou

**Affiliations:** 1 State Key Laboratory of Membrane Biology and Beijing Key Laboratory of Cardiometabolic Molecular Medicine, Institute of Molecular Medicine and Peking-Tsinghua Center for Life Sciences and PKU-IDG/McGovern Institute for Brain Research, Peking University, Beijing, 100871 China; 2 Center for Mitochondrial Biology and Medicine, The Key Laboratory of Biomedical Information Engineering of Ministry of Education, School of Life Science and Technology and Frontier Institute of Science and Technology, Xi’an Jiaotong University, Xi’an, 710049 China; 3 College of Life Sciences, Liaocheng University, Liaocheng, 252059 China; 4 Key Lab of Medical Electrophysiology, Ministry of Education, Institute of Cardiovascular Research, Southwest Medical University, Luzhou, 646000 China; 5 Departments of Neurology and Neuroscience, Friedman Brain Institute, Icahn School of Medicine at Mount Sinai, New York, NY 10029 USA

## Abstract

Loss-of-function mutations in *Parkin* are the most common causes of autosomal recessive Parkinson’s disease (PD). Many putative substrates of parkin have been reported; their pathogenic roles, however, remain obscure due to poor characterization, particularly in vivo. Here, we show that synaptotagmin-11, encoded by a PD-risk gene *SYT11*, is a physiological substrate of parkin and plays critical roles in mediating parkin-linked neurotoxicity. Unilateral overexpression of full-length, but not C2B-truncated, synaptotagmin-11 in the substantia nigra pars compacta (SNpc) impairs ipsilateral striatal dopamine release, causes late-onset degeneration of dopaminergic neurons, and induces progressive contralateral motor abnormalities. Mechanistically, synaptotagmin-11 impairs vesicle pool replenishment and thus dopamine release by inhibiting endocytosis. Furthermore, parkin deficiency induces synaptotagmin-11 accumulation and PD-like neurotoxicity in mouse models, which is reversed by *SYT11* knockdown in the SNpc or knockout of *SYT11* restricted to dopaminergic neurons. Thus, PD-like neurotoxicity induced by parkin dysfunction requires synaptotagmin-11 accumulation in SNpc dopaminergic neurons.

## Abstract (web summary)

Mutations in the parkin, an ubiquitin ligase, are linked to Parkinson’s disease. Here the authors show that synaptotagmin-11 is a parkin substrate and that its upregulation affects dopamine release, triggers degeneration, and causes motor impairment.

## Introduction

Parkinson’s disease (PD) is a complex neurodegenerative movement disorder characterized by the selective vulnerability of dopaminergic neurons in the substantia nigra pars compacta (SNpc), and the impairment of dopamine (DA) release in the striatum^1–7^. Numerous PD-risk genes and environmental factors have been identified, but their functional connectivity is poorly understood, thus hindering the elucidation of a common pathogenic pathway for PD. Parkin (encoded by *PARK*2) is an E3 ubiquitin ligase that regulates proteasome-dependent protein degradation^1,3,8,9^ and mitochondrial autophagy (mitophagy)^10–13^. It has been shown that mutations in parkin affect either its E3 ligase activity or its interactions with E2 enzymes, causing familial recessive and sporadic early-onset PD^1,8,12,14–17^. Abnormal accumulation of parkin substrates may contribute to DA neuron degeneration in PD associated with parkin mutations^5^. Although many putative substrates of parkin have been identified^1–3,10,18–20^, due to a lack of unequivocal in vivo evidence, the involvement of these substrates in the pathogenesis of PD and the underlying mechanisms remain elusive.

A recent meta-analysis of PD populations based on five genome-wide association studies has identified 11 loci linked to a significant risk of PD, including synaptotagmin-11 (*SYT11*) as one of five novel PD-risk genes^21^. A number of Syt proteins have been shown to be Ca^2+^ sensors for SNARE-mediated vesicle fusion during neurotransmitter release and hormone secretion^22^. Syt1 and Syt4 have also been shown to function in endocytosis^23–27^. We recently found that Syt11, a non-Ca^2+^-binding Syt^28^, inhibits endocytosis and thus vesicle recycling in neurons^29^. Its role in neurodegenerative disease, however, has never been reported. Interestingly, Syt11 can be ubiquitinated by parkin in vitro and accumulates in Lewy bodies in the sporadic human PD patient brain tissue^19^. Here we show that Syt11 is a parkin substrate and mediates PD-like neurotoxicity and behavioral deficits.

## Results

### Syt11 is a parkin substrate

**Fig. 1:**
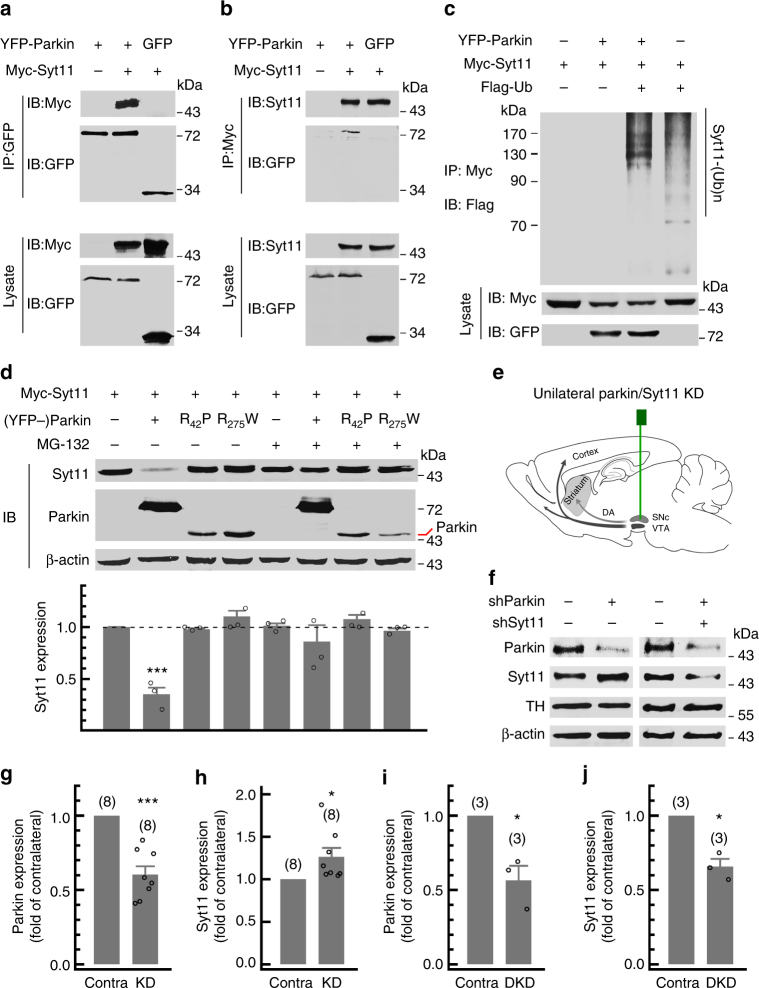
Effect of Parkin dysfunction on Syt11 accumulation. **a**, **b** Co-immunoprecipitation of Syt11 with parkin. YFP-parkin (or GFP) and Myc-Syt11 was co-expressed in HEK293 cells, and the lysates were immunoprecipitated (IP) with anti-GFP or anti-Myc antibodies, followed by immunoblotting (IB) with antibodies as indicated. Whole-cell lysates were also immunoblotted with the indicated antibodies as controls. **c** Parkin catalyzes the ubiquitination of Syt11. Ubiquitination of Syt11 in HEK293 cells expressing combinations of Myc-Syt11, YFP-parkin, and Flag-ubiquitin (Flag-Ub) as indicated. Cell lysates were immunoprecipitated with anti-Myc, followed by immunoblotting with anti-Flag antibody. **d** Parkin mediates proteasome-dependent degradation of Syt11. Immunoblotting for Syt11 in HEK293 cells expressing YFP-parkin or parkin mutants (R42P disrupting the ubiquitin-like domain, or R275W disrupting the RING1 domain of parkin), with or without 12 h treatment with MG132 (10 μM). Data were collected from three independent experiments (*P* = 0.041 for ANOVA, *P* < 0.001 for further LSD test between Myc-Syt11/YFP-parkin and Myc-Syt11). **e** Cartoon of virus injection for unilateral parkin knockdown (KD) and parkin/syt11 double-KD in the SNpc. **f** Representative western blots showing the expression of parkin, Syt11, and TH in the bilateral SNpc from parkin KD and parkin/syt11 double-KD mice 1 month after virus injection as in **e**. **g**–**j** Statistics of parkin and Syt11 expression in the SNpc from mice as indicated (*P* = 0.000, 0.040, 0.047, and 0.022; number in parentheses represents number of mice/biological repeats). Data are shown as mean ± s.e.m. One-way ANOVA for **d** and paired Student’s *t*-test for **g**–**j**, *P *<* 0.05, ***P *<* 0.001

To determine whether Syt11 is a parkin substrate, we used co-immunoprecipitation and found an interaction between Syt11 and parkin in HEK293 cells overexpressing YFP-parkin and Myc-Syt11 (Fig. 1a, b). We next verified that parkin ubiquitinates Syt11 in vitro^19^. The analysis of anti-Myc immunoprecipitates from the lysates of HEK293 cells transfected with Myc-Syt11, YFP-parkin, and FLAG-ubiquitin (Flag-Ub) revealed that the ubiquitination of Syt11 was markedly increased by the overexpression of parkin (Fig. 1c). In addition, Syt11 expression was greatly reduced by parkin overexpression, while the pathogenic parkin mutations found in PD patients^8,10,30,31^, such as R42P (disrupting the ubiquitin-like domain) and R275W (disrupting the RING1 domain), failed to mediate Syt11 degradation (Fig. 1d). Furthermore, the parkin-mediated decrease of Syt11 level was prevented by 12-h pretreatment with the proteasome inhibitor MG132 (Fig. 1d), confirming that parkin-mediated Syt11 degradation occurs through the proteasome pathway.

To further confirm that parkin also regulates endogenous Syt11 expression, we overexpressed YFP-parkin in hippocampal neurons and collected the transfected cells with a fluorescence-activated cell sorter. Western blot analysis revealed that the endogenous Syt11 expression in primary neurons was also decreased by parkin overexpression (Supplementary Fig. 1). Importantly, knockdown (KD) of parkin expression using a lentivirus carrying parkin shRNA (shParkin) induced the accumulation of Syt11 in the SNpc (Fig. 1e–j and Supplementary Fig. 12a–c), while the scrambled shRNA served as a control (parkin_Ipsi/Contra_: 1.01 ± 0.24, *P* = 0.97; Syt11_Ipsi/Contra_ = 1.02 ± 0.05, *P* = 0.73; *n* = 3), verifying the role of parkin in regulating Syt11 levels in vivo. Together with the previous in vitro data^19^, our findings demonstrate that Syt11 is a parkin substrate, while parkin acts as the E3 ligase to directly regulate Syt11 protein levels through ubiquitin-dependent proteasome degradation.

### Syt11 accumulation induces behavioral deficits

**Fig. 2:**
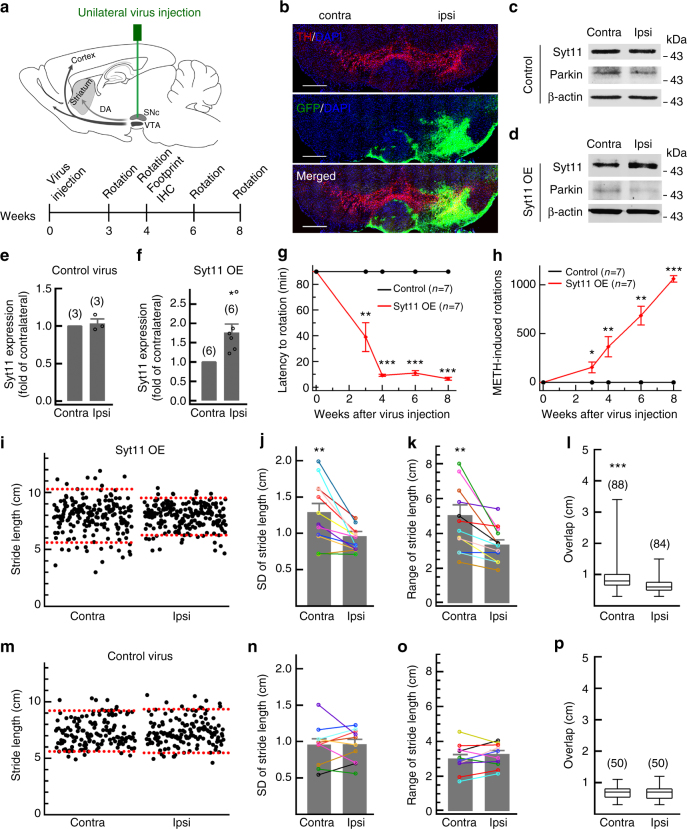
Unilateral Syt11 overexpression in the SNpc induces motor abnormalities. **a** Schematic of unilateral virus injection in the SNpc and time course of experiments. **b** Representative micrograph of TH staining in an SNpc-containing slice showing that GFP-carrying lentivirus was precisely injected into the SNpc (ipsi) and efficiently infected the dopaminergic neurons. Scale bars, 500 μm. **c**–**f** Representative western blots and statistics showing the expression of Syt11 in the bilateral SNpc from mice overexpressing (OE) Syt11 unilaterally and controls 1 month after virus injection (Control, *P* *=* 0.691; Syt11-OE, *P* *=* 0.022). **g**,** h** Methamphetamine (METH) induces progressive asymmetric rotation (rotation latency and rotations in 90 min) of mice with unilateral OE of Syt11 in the SNpc, compared with control virus (latency, *P* *=* 0.022, 0.000, 0.000, and 0.000 for 3, 4, 6, and 8 weeks; rotation, *P* *=* 0.020, 0.007, 0.003, and 0.000 for 3, 4, 6, and 8 weeks). **i**–**l** Footprint data showing the defect in contralateral motor stability of mice with unilateral Syt11-OE in the SNpc. Stride length (**i**–**k**, SD, *P* *=* 0.003; range, *P* *=* 0.004) and front/hind footprint overlap (**l**, *P* *<* 0.001, *n* = 88 Contra and 84 Ipsi steps from six mice) were quantified, and the dashed red lines in **i** represent the 5 and 95% percentiles of stride length. **m**–**p** Footprint data showing the normal motor stability of mice unilaterally expressing control virus in the SNpc (SD, *P* *=* 0.946; range, *P* *=* 0.347; foot overlap, *P* *=* 0.857, *n* = 50 Contra and 50 Ipsi steps from six mice). Contra, contralateral; Ipsi, ipsilateral. Data are shown as mean ± s.e.m. for **e**–**h**, **j**, **k**, **n**, **o**. Paired Student’s *t*-test for **e**, **f**,** j**, **k**, **n**, **o**; unpaired Student’s *t*-test for **g**, **h**; Mann–Whitney test for **l**, **p**, box and whisker plots show medians (central line in the box), ranges between 25th and 75th percentiles (box) and minimum–maximum ranges (whiskers). *P *<* 0.05, **P *<* 0.01, ***P *<* 0.001

Since parkin deficiency and its pathogenic mutations induce the abnormal accumulation of Syt11 (Fig. 1), we next asked whether increasing Syt11 levels alone is sufficient to trigger PD-related toxicity in the mouse brain. Syt11-carrying lentivirus was delivered to the SNpc in the right hemisphere by stereotaxic injection (Fig. 2a). The results showed efficient infection of dopaminergic neurons in the SNpc, as evidenced by GFP expression in tyrosine hydroxylase (TH)-positive cells (Fig. 2b). Parkin expression remained largely unchanged in mice overexpressing Syt11 (Fig. 2d and Supplementary Fig. 2). Since Syt11 was unilaterally overexpressed in the SNpc region, we used methamphetamine (METH)-induced rotational asymmetry^20,32^ as a functional behavioral readout to assess the progression and severity of motor abnormalities induced by Syt11 overexpression. Mice injected with virus carrying GFP-only served as controls and they showed no defect of asymmetric rotation (Fig. 2c–h). Strikingly, unilateral overexpression (OE) of Syt11 induced a progressive increase in METH-induced contralateral rotation and a gradually shortened latency to rotation during 3–8 weeks after virus injection (Fig. 2c–h). Footprint gait analysis^33,34^, an assay that does not depend on pharmacological stimulation, also showed an unbalanced gait in Syt11-OE mice 1 month after virus injection (Fig. 2i–l). Mice with unilateral Syt11-OE showed greater variation of the contralateral stride length (Fig. 2i), which was similar on average to that of the ipsilateral side (contralateral, 7.93 ± 0.22; ipsilateral, 7.85 ± 0.18, *P* = 0.23), but with a larger standard deviation and range (Fig. 2j, k). Consistently, Syt11-OE mice also showed greater contralateral than ipsilateral front/hindpaw overlap (Fig. 2l). In contrast, both stride length and paw overlap were similar on both sides of the control virus-injected mice (Fig. 2m–p). Collectively, these findings indicate that Syt11 overexpression in this brain region is sufficient to induce locomotor deficits.

### Syt11 accumulation impairs DA release

**Fig. 3:**
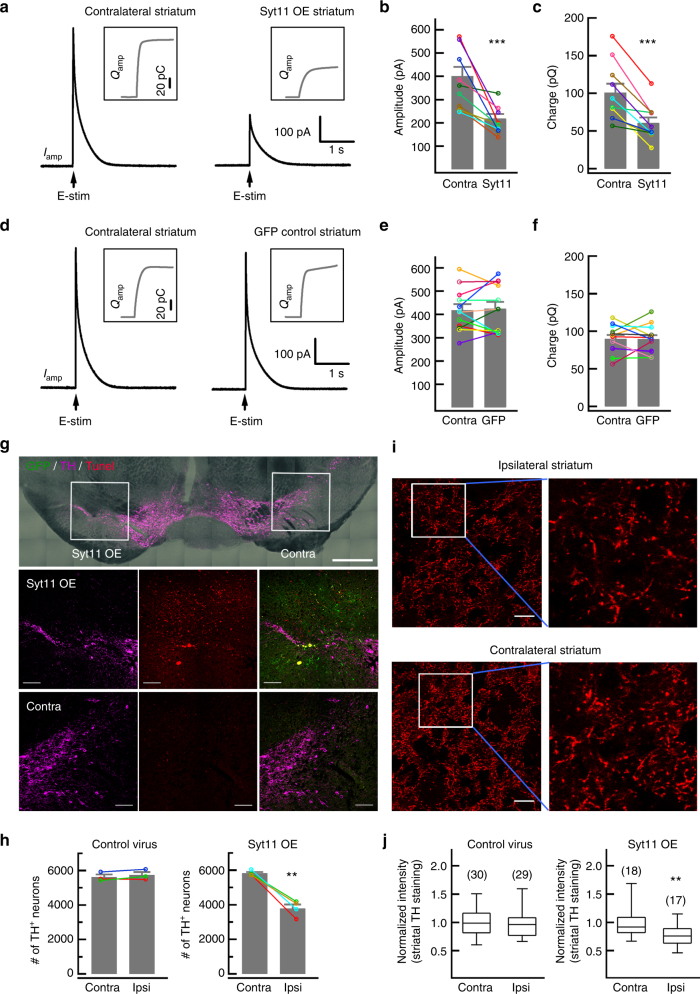
Syt11 overexpression induces defects in striatal DA release and loss of dopaminergic neurons in the SNpc. **a**–**c** Representative amperometric currents (*I*_amp_) and statistics showing reduced DA release from Syt11-overexpressing (OE) dopaminergic terminals in the striatum. Syt11-carrying lentivirus was unilaterally injected into the SNpc and CFE recordings were made in striatal slices on both sides to compare the contralateral and ipsilateral (Syt11-OE) DA release (*P* *<* 0.001, *n* = 10 pairs in slices from four mice). Insets: cumulative traces (charge) of *I*_amp_. **d**–**f** Normal striatal DA release from mice infected by control GFP-carrying lentivirus in the SNpc (amplitude, *P* *=* 0.730; charge, *P* *=* 0.947; *n* = 12 pairs in slices from 5 mice). **g**, **h** Syt11-OE induces apoptosis of DA neurons in the SNpc. Syt11-carrying lentivirus was unilaterally injected into the SNpc and dopaminergic neurons with TH staining were counted on both sides 3 months later (Control, *P* *=* 0.268; Syt11-OE, *P* *=* 0.004). TUNEL staining was used to detect apoptosis of dopaminergic neurons in the Syt11-OE SNpc (*n* = 3 control and 4 Syt11-OE mice, biological repeats). **i**, **j** Unilateral Syt11-OE in the SNpc reduces the density of TH-positive fibers in the ipsilateral striatum. The normalized fluorescence intensity of TH staining in the striatum is shown in **j** (Control, *P* *=* 0.624; Syt11-OE, *P* *=* 0.009). Data were collected from three control mice and four Syt11-OE mice. Scale bars, 500 μm (upper panel) and 100 μm (middle and lower panels) in **g**, 20 μm in **i**. Data are shown as mean ± s.e.m. and paired Student’s *t*-test is used for **b**,** c**, **e**, **f**,** h**. Mann–Whitney test is used for **j**, box and whisker plots show medians (central line in the box), ranges between 25th and 75th percentiles (box) and minimum–maximum ranges (whiskers). **P *<* 0.01, ***P *<* 0.001

We next made amperometric recordings with electrochemical carbon fiber electrodes (CFEs) in striatal slices to determine whether Syt11-OE in the SNpc impairs DA release from nigrostriatal terminals^32,35^. When a local electrical stimulus (Estim) was applied to the striatal slice, there was a transient increase in amperometric current (*I*_amp_, with an amplitude of ~400 pA) with a subsequent decay to baseline, representing a transient increase in extracellular DA concentration (~2.4 μM, Fig. 3a and Supplementary Fig. 3). Strikingly, unilateral overexpression of Syt11 in the SNpc markedly decreased the DA release in the ipsilateral striatum compared with that in the contralateral striatum of the same mice at 1 month after virus injection (Fig. 3a–c). In contrast, DA release in the ipsilateral striatum remained intact when control virus was used (Fig. 3d–f). In addition, Syt11-OE inhibited secretion in neuroendocrine adrenal chromaffin cells (Supplementary Fig. 4). Together, these results demonstrate that Syt11 overexpression in the SNpc in vivo is sufficient to cause a defect of DA release in the striatum.

### Syt11 accumulation leads to late onset loss of DA neurons

To investigate whether Syt11 overexpression also leads to the loss of dopaminergic neurons in the SNpc, we performed stereological counting of TH-positive neurons in the Syt11-OE and contralateral sides of the SNpc^20,33^. Interestingly, Syt11-OE failed to induce a clear loss of dopaminergic neurons in the SNpc at one month after injection (Supplementary Fig. 5), despite the robust motor impairment (Fig. 2) and DA release defects (Fig. 3a–f) at this stage. However, we observed ~35% loss of DA neurons in the SNpc with Syt11-OE at 3 months after injection, while no significant loss of TH-positive neurons was found when control virus was used (Fig. 3g, h). We also found positive terminal deoxynucleotidyl transferase dUTP nick end labeling (TUNEL) staining in the Syt11-OE, but not in the contralateral SNpc (Fig. 3g), suggesting that these DA neurons degenerate through apoptosis. Consistently, the reduced fluorescence intensity of TH staining also revealed a substantial reduction of TH-positive neurites in the ipsilateral striatum (Fig. 3i, j). Thus, our data indicate that Syt11 overexpression also induces the progressive loss of dopaminergic neurons, which is preceded by depression of DA release.

### Syt11 inhibits endocytosis and vesicle replenishment

**Fig. 4:**
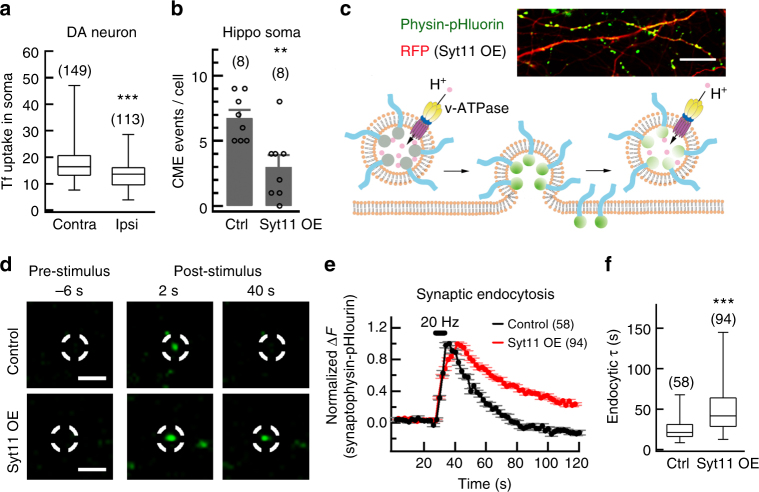
Syt11 inhibits endocytosis in neurons. **a** Statistics showing the decreased transferrin (Tf) uptake of dopaminergic neurons in the SNpc by Syt11 overexpression (OE). Syt11-carrying lentivirus was unilaterally injected into the SNpc in TH-GFP mice and Tf uptake was assessed in the contralateral (Contra) and Syt11-overexpressing (Ipsi, ipsilateral) sides of the SNpc 1 month later (*P* *<* 0.001, *n* represents number of neurons). **b** TIRF imaging of Clathrin-DsRed showing the inhibitory role of Syt11 in clathrin-mediated endocytosis (CME) in the somata of hippocampal neurons (Hippo soma, data are shown as mean ± s.e.m., *P* *=* 0.010, *n* represents number of imaged neurons). **c** Representative micrograph and cartoon showing the real-time imaging of synaptophysin-pHluorin during exocytosis and endocytosis. Upper panel, confocal image showing the expression of physin-pHluorin in hippocampal neurons. Lower panel, cartoon showing the fluorescence change of physin-pHluorin during exocytosis and endocytosis. **d** Sample images showing phsin-pHluorin fluorescence in presynaptic boutons of control (RFP-only) or Syt11 overexpressing (OE) hippocampal neurons before, 2 s after and 40 s after 100 stimuli at 20 Hz. **e**, **f** Normalized fluorescence changes and statistics of endocytic tau (*τ*) of synaptophysin-pHluorin in Syt11 overexpressing or control hippocampal synapses (*P* *<* 0.001, *n* represents the number of imaged synaptic boutons). Scale bars, 20 μm for **c** and 5 μm for **d**. Unpaired Student’s *t*-test for **b**, Mann–Whitney test for **a**, **f**, **d**, box and whisker plots show medians (central line in the box), ranges between 25th and 75th percentiles (box) and minimum–maximum ranges (whiskers). **P *<* 0.01, ***P *<* 0.001

An independent study from our lab has shown that syt11 inhibits dynamin-dependent endocytosis by limiting the membrane invagination in dorsal root ganglion and hippocampal neurons^27,29^, so we next determined whether the impaired DA transmission was also due to the inhibitory role of Syt11 in endocytosis in dopaminergic neurons. Alexa Fluor-conjugated transferrin uptake was measured to evaluate clathrin-mediated endocytosis (CME) in Syt11-overexpressing dopaminergic neurons in the SNpc of TH-driven GFP transgenic mice^29,36^. We found that, compared with the contralateral side, transferrin uptake was markedly suppressed in TH-positive neurons in the Syt11-OE SNpc (Fig. 4a and Supplementary Fig. 6), indicating that CME was slowed due to Syt11-OE. To further verify the inhibitory role of Syt11 in neuronal endocytosis, we used total internal reflection fluorescence (TIRF) imaging of clathrin-DsRed in the somata of hippocampal neurons with or without Syt11 overexpression. Strikingly, most of the assembled clathrin clusters were clamped at the plasma membrane, and thus the CME events were markedly reduced in Syt11-OE neurons (Fig. 4b and Supplementary Fig. 7). Furthermore, we used live imaging of the synaptophysin-pHluorin reporter, an assay for stimulated synaptic endocytosis/exocytosis (Fig. 4c), to monitor the function of Syt11 in endocytosis and vesicle recycling. Consistently, Syt11-OE neurons showed a robust reduction in endocytic rate with a significantly longer time constant, confirming the inhibitory role of Syt11 in synaptic endocytosis and vesicle recycling of hippocampal neurons (Fig. 4d–f). These findings indicate an inhibitory action of Syt11 on endocytosis in DA neurons.

**Fig. 5:**
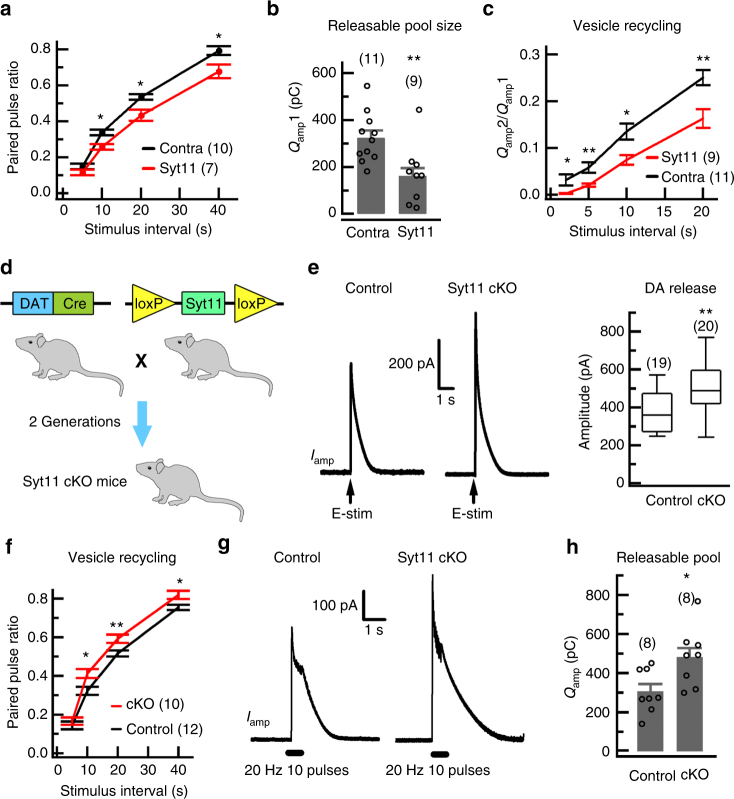
Syt11 inhibits vesicle pool replenishment in dopaminergic neurons. **a** Paired-pulse ratios of DA release with different interstimulus intervals recorded in the striatum contralateral (Contra) or ipsilateral (Syt11) to the SNpc with Syt11-OE (*P* *=* 0.075, 0.021, 0.016, and 0.022 for 5, 10, 20, and 40 s; *n* represents slices from four mice). **b** Statistics showing the reduced releasable vesicle pool size (*Q*_amp_1) by Syt11-OE revealed by the DA release in response to a burst of pulses (20 Hz, 10 pulses). *P* *=* 0.003, *n* represents slices from four mice. **c** Recovery rate of readily-releasable vesicle pools (*Q*_amp_2/*Q*_amp_1) at different interstimulus intervals. A burst of pulses (20 Hz, 10 pulses) was used to deplete the releasable vesicle pools (*Q*_amp_1) and the following single-pulse stimulation was used to assess the recovery of readily-releasable vesicle pools (*Q*_amp_2). *P* *=* 0.026, 0.007, 0.012, and 0.007 for 2, 5, 10, and 20 s; *n* represents slices from four mice. **d** Schematic of the generation of DAT-driven Syt11-cKO mice. Homozygous floxed Syt11-null mice were crossed with DA transporter-driven Cre heterozygous knockin (DAT-Cre) mice to produce DA neuron-restricted Syt11 conditional knockout (Syt11-cKO) mice. **e** Representative *I*_amp_ evoked by single Estim pulse and statistics showing increased DA release in the striatum of Syt11-cKO mice (*P* *=* 0.008, *n* represents slices from five mice). **f** Paired-pulse ratios of DA release with different interstimulus intervals (*P* *=* 0.443, 0.010, 0.006, and 0.017 for 5, 10, 20, and 40 s; *n* represents slices from five mice). **g** Representative *I*_amp_ evoked by burst Estim pulses in the striatum of control and Syt11-cKO mice. **h** Statistics showing the increased releasable vesicle pool size (*Q*_amp_) in the striatum in Syt11-cKO mice (*P* *=* 0.020, *n* represents slices from four control and three Syt11-cKO mice). Data are shown as mean ± s.e.m. and unpaired Student’s *t*-test is used for **a**–**c**,** f**, **h**. Mann–Whitney test is used for **e**, box and whisker plots show medians (central line in the box), ranges between 25th and 75th percentiles (box) and minimum–maximum ranges (whiskers). *P *<* 0.05, **P *<* 0.01

DA release in response to paired-pulse stimulation was used to further assess the role of Syt11 in vesicle recycling in DA neurons. As expected, we found a reduced paired-pulse ratio of DA release in ipsilateral striatal slices overexpressing Syt11 (Fig. 5a and Supplementary Fig. 8), indicating that the vesicle replenishment^35^ was also inhibited. Burst stimulation using a train of 10 pulses at 20 Hz also revealed a reduced releasable vesicle pool in the striatal DA terminals on the Syt11-OE side (Fig. 5b and Supplementary Fig. 9a–c). In addition, we re-examined the vesicle recycling rate after depleting the releasable vesicle pools and confirmed the slowed vesicle replenishment by Syt11 (Fig. 5c and Supplementary Fig. 9). Furthermore, we crossed homozygous floxed Syt11-null mice with heterozygous DA transporter-driven Cre-knockin mice (DAT-Cre)^37–39^ to produce DA neuron-restricted Syt11 conditional knockout (Syt11-cKO) mice (Fig. 5d). As expected, immunostaining showed a nearly complete loss of Syt11 in TH-positive neurons in the ventral midbrain, while Syt11 expression in TH-negative neurons remained intact (Supplementary Fig. 10a, b), indicating a specific knockout of Syt11 in dopaminergic neurons. Consistently, western blots showed a dramatic reduction of Syt11 expression in the whole ventral midbrain (Supplementary Fig. 10c–e). Strikingly, amperometric recordings demonstrated increased DA release (Fig. 5e), accelerated vesicle replenishment (Fig. 5f), and enlarged releasable vesicle pools (Fig. 5g, h) in the striatum of Syt11-cKO mice, confirming the inhibitory role of Syt11 in vesicle recycling in DA neurons.

**Fig. 6:**
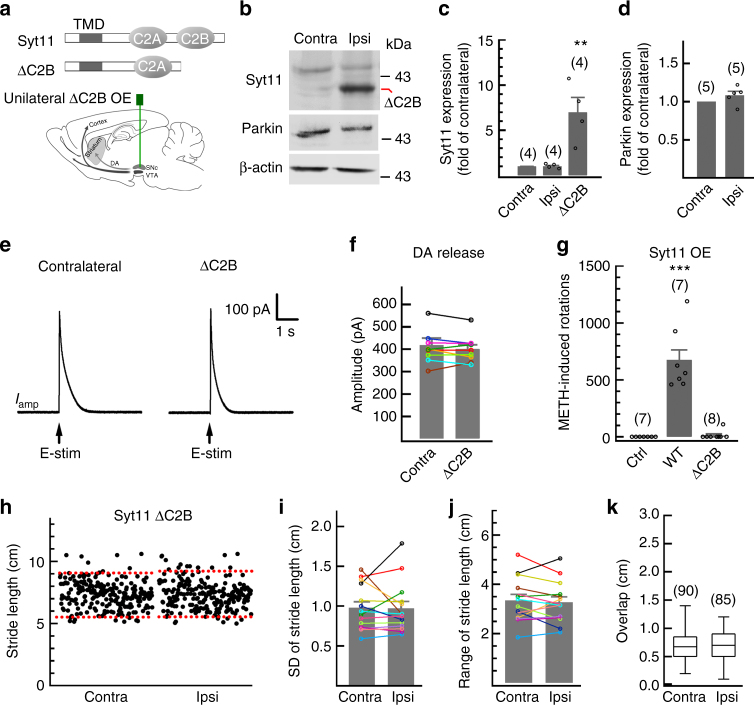
Overexpression of C2B-truncated Syt11 in the SNpc fails to mediate deficits in DA release and motor behavior. **a** Schematic of unilateral overexpression of C2B-truncated Syt11 (ΔC2B) in the SNpc with an AAV9-expressing system. **b** Representative western blots showing the expression of parkin and Syt11 in the bilateral SNpc from mice with unilateral overexpression of ΔC2B as in **a**. Western blots were performed 1 month after virus injection. **c**, **d** Statistics of Syt11 (*P* = 0.002) and parkin (*P* = 0.196) expression in the SNpc from mice as in **b**. **e**, **f** Representative amperometric currents (*I*_amp_) and statistics showing the intact DA release from ΔC2B-overexpressing dopaminergic terminals in the striatum (*P* = 0.332,* n* = 9 pairs in slices from five mice). **g** METH-induced asymmetric rotations (in 90 min) of control (Ctrl), full-length Syt11 (WT), and ΔC2B-Syt11 overexpressing mice (*P* < 0.001). **h**–**k** Footprint data showing that unilateral overexpression of ΔC2B in the SNpc fails to induce a motor defect in the contralateral limbs (SD, *P* = 0.900; range, *P* = 0.516; overlap, *P* = 0.917, *n* = 90 Contra and 85 Ipsi steps from 7 mice). Dashed red lines show the 5 and 95% percentiles of stride length in **h**. Data are shown as mean ± s.e.m for **c**, **d**, **f**, **g**, **i**, **j**. Paired Student’s *t*-test for **d**, **f**, **i**, **j**; one-way ANOVA for **c**, **g**; Mann–Whitney test for **k**, box and whisker plots show medians (central line in the box), ranges between 25th and 75th percentiles (box) and minimum–maximum ranges (whiskers). *P *<* 0.05, **P *<* 0.01, ***P *<* 0.001

Structure–function analysis revealed that the C2B domain of Syt11 is critical for its inhibitory role in endocytosis^29^. We next investigated whether this domain is also essential for Syt11 to mediate PD-related neurotoxicity (Fig. 6a). Strikingly, unilateral overexpression of the C2B-truncated form of Syt11 in the SNpc (Fig. 6b–d) failed to impair the ipsilateral striatal DA release (Fig. 6e, f) and the contralateral motor stability (Fig. 6g–k), indicating the critical role of the C2B domain in the Syt11-mediated pathogenesis of PD. Collectively, these findings suggest that Syt11 plays an important role in DA transmission by regulating endocytosis and the vesicle-recycling process, while the upregulation/accumulation of Syt11 causes the reduction of releasable vesicle pools, impairment of DA release in the striatum, and thus the progression of PD.

### Syt11 is essential for the parkin-linked neurotoxicity

**Fig. 7:**
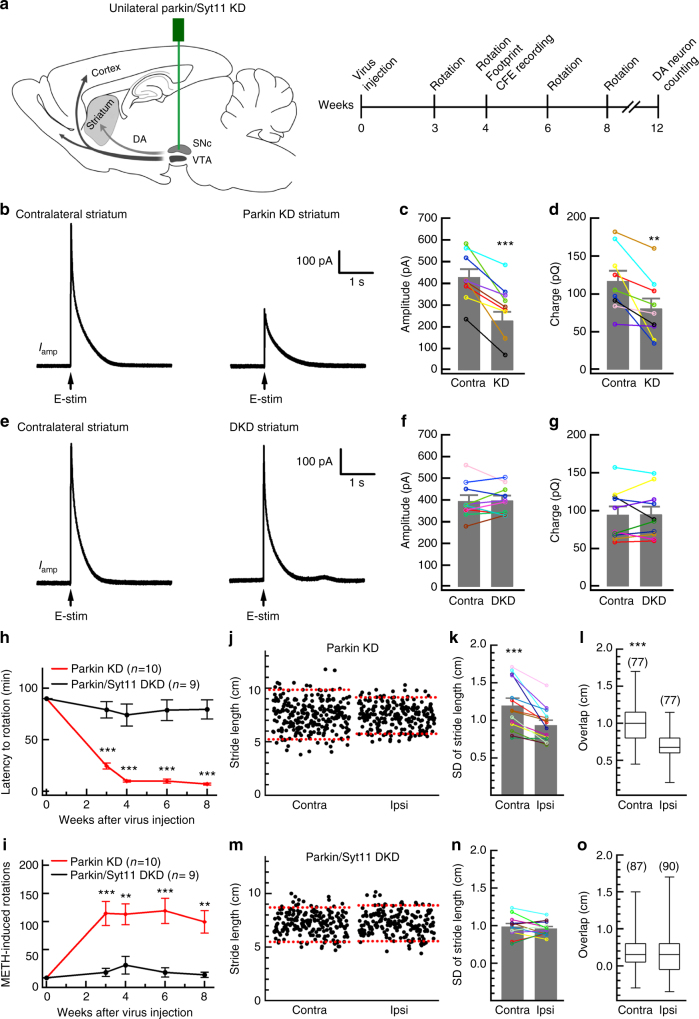
Syt11 knockdown rescues parkin knockdown-induced deficits in DA release and motor behavior. **a** Schematic of unilateral parkin knockdown (KD) and parkin/syt11 double-KD in the SNpc and time course experiments. **b**–**d** Unilateral KD of parkin in the SNpc reduces DA release in the ipsilateral striatum. Statistics of the amplitude and charge of DA release from the bilateral striatum (amplitute, *P* *=* 0.001; charge, *P* *=* 0.008; *n* = 9 pairs in slices from five mice). **e**–**g** Syt11 KD reverses the reduction of DA release by parkin KD in the ipsilateral striatum (amplitute, *P* *=* 0.829; charge, *P* *=* 0.916; *n *= 10 pairs in slices from six mice). **h**, **i** METH-induced asymmetric rotation (latency and rotations in 90 min) in mice with unilateral parkin KD or parkin/Syt11 double-KD in the SNpc (latency, *P* *<* 0.001 for all time points; rotation, *P* *=* 0.001, 0.005, 0.000, and 0.002 for 3, 4, 6, and 8 weeks). **j**–**o** Footprint data showing that Syt11 KD reverses the parkin KD-induced motor defect in the contralateral limbs; dashed red lines show the 5 and 95% percentiles of stride length in **j** and **m**. *n* = 77 Contra and 77 Ipsi steps from seven parkin KD mice (*P* *<* 0.001); *n* = 87 Contra and 90 Ipsi steps from six DKD mice (SD, *P* *=* 0.420; Overlap, *P* *=* 0.797). Data are shown as mean ± s.e.m for **c**–**g**, **k**, **n**. Contra, contralateral; Ipsi, ipsilateral. Paired Student’s *t*-test for **c**, **d**, **f**, **g**, **k**, **n**; unpaired Student’s *t*-test for **h**, **i**; Mann–Whitney test for **l**, **o**, box and whisker plots show medians (central line in the box), ranges between 25th and 75th percentiles (box) and minimum–maximum ranges (whiskers). *P *<* 0.05, **P *<* 0.01, ***P *<* 0.001

Considering the possible developmental compensation in parkin-KO mice^17,20,40,41^, to test the idea that Syt11 accumulation mediates the pathogenic role of parkin in PD, we knocked down both *Syt11* and *Parkin* expression. We co-injected lentivirus expressing shParkin and lentivirus expressing Syt11 shRNA (shSyt11) or shRNA control (1:1 ratio) unilaterally into the SNpc (Fig. 7a). The combination of the two GFP- and RFP-expressing scrambled shRNA control lentiviruses showed high co-infection efficiency (>80%) in the midbrain in vivo (Supplementary Fig. 11). Immunoblotting showed the abnormal accumulation of Syt11 in the shParkin (plus control virus)-injected SNpc (Fig. 1e–h and Supplementary Fig. 12a–c). Co-injection of virus carrying shParkin and shSyt11 abolished the accumulated Syt11 expression due to parkin KD only (Fig. 1e–j). Consistent with the impaired DA release in parkin-KO mice^42^, amperometric recording showed the decreased nigrostriatal DA release caused by parkin KD in the SNpc at 1 month after injection (Fig. 7b–d and Supplementary Fig. 12d, e). Interestingly, additional Syt11 KD completely reversed the parkin KD-induced impairment of striatal DA release (Fig. 7e–g). These results demonstrated the critical role of Syt11 accumulation in the reduced DA release caused by parkin deficiency. Furthermore, TH staining analysis showed that parkin KD induced a remarkable loss of DA neurons in the SNpc, which was reversed by parkin/Syt11 double-KD at 3 months after virus injection (Supplementary Fig. 13). METH induced progressive asymmetric rotation in mice with unilateral KD of parkin in the SNpc during 8 weeks after virus injection, and this abnormal motor activity was attenuated in mice with double-KD of parkin and Syt11 (Fig. 7h, i and Supplementary Fig. 12f). In addition, the impaired contralateral motor stability also occurred in mice with unilateral parkin KD in the SNpc (Fig. 7j–l and Supplementary Fig. 12g–j), but was prevented by Syt11/parkin double-KD (Fig. 7m–o). These results establish that Syt11 accumulation is critical for parkin to mediate PD-related neurotoxicity.

**Fig. 8:**
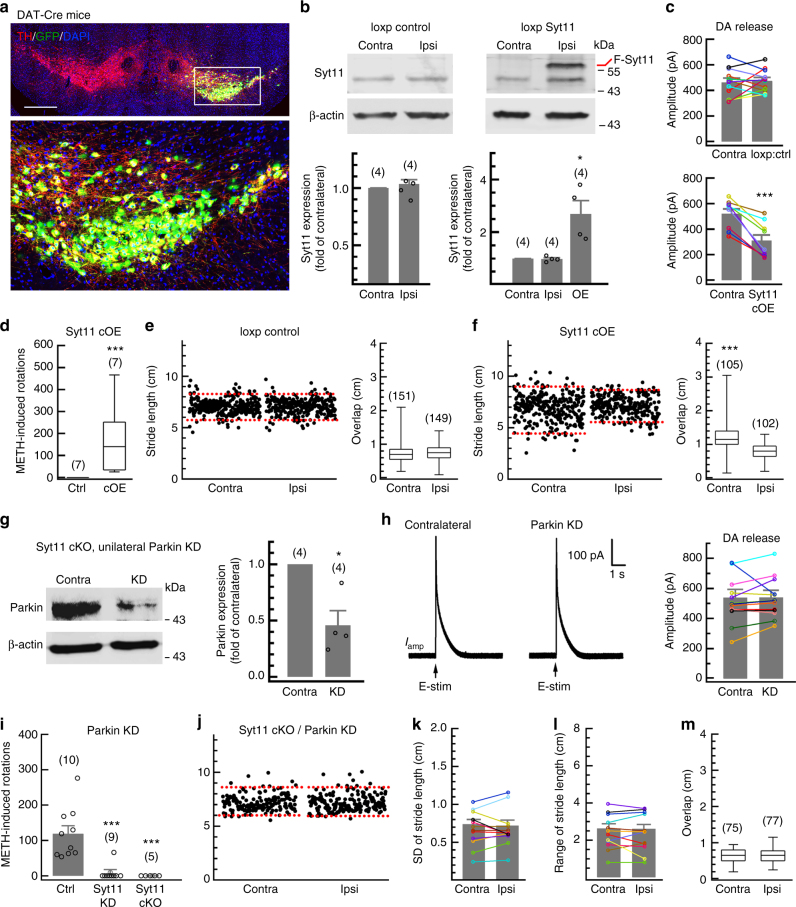
Expression of Syt11 in dopaminergic neurons is critical for parkin to mediate DA release and motor deficits. **a** Representative micrograph of TH staining in an SNpc-containing slice from DAT-Cre mice unilaterally injected with Cre-inducible Flag-Syt11 (or GFP control) overexpressing AAV9 virus. Scale bar, 500 μm. **b** Representative western blots and statistics showing the expression of Syt11 and Flag-Syt11 (F-Syt11, OE) in the bilateral SNpc from Syt11 cOE mice 1 month after virus injection. **c** Statistics of DA release in the striatum of Syt11 cOE mice (*n* = 12 slices from five control mice and nine slices from four Syt11 cOE mice). **d** METH-induced asymmetric rotation (in 90 min) of control (Ctrl) and Syt11 cOE mice. **e**, **f** Footprint gait analysis of Syt11 cOE and loxp control mice; dashed red lines show the 5 and 95% percentiles of stride length. *n* represents steps from seven control (**e**) or six Syt11 cOE mice (**f**). **g** Representative western blots and statistics showing the efficiency of parkin KD in the SNpc of Syt11-cKO mice. **h** Representative amperometric currents and statistics showing the unchanged DA release in the striatum of Syt11-cKO mice with unilateral parkin KD in the SNpc (*n* = 13 pairs in slices from five mice). **i** METH-induced asymmetric rotation of control mice with unilateral parkin KD or parkin/Syt11 double-KD in the SNpc, and that of Syt11-cKO mice with unilateral parkin KD. **j**–**m** Footprint data showing that unilateral parkin KD in the SNpc fails to induce a motor defect in the contralateral limbs of Syt11-cKO mice (*n* = 75 Contra and 77 Ipsi steps from five mice). Dashed red lines show the 5 and 95% percentiles of stride length in **j**. Data are shown as mean ± s.e.m for **b**, **c**, **g**–**l**. Paired Student’s *t*-test for **b** lower left, **c**, **g**, **h**, **k**, **l**; one-way ANOVA for **b** lower right, **i**; Mann-Whitney test for **d**–**f**, **m**, box and whisker plots show medians (central line in the box), ranges between 25th and 75th percentiles (box) and minimum–maximum ranges (whiskers). *P *<* 0.05, ***P *<* 0.001

Since the PD-related neurotoxicity could be due to the direct defects of the SNpc DA neurons or indirect effects of other cells in this region, we sought to determine whether the Syt11 accumulation in DA neuron is the major cause of the neuronal toxicity in PD. We unilaterally overexpressed Syt11 in SNpc DA neurons by injecting a FLEx^loxP^-based Flag-Syt11-overexpressing AAV9 virus into the right SNpc of DAT-Cre mice. Virus expressing FLEx^loxP^-based GFP only served as a control (loxp control, Fig. 8a–f). TH staining showed specific overexpression of Syt11 (or GFP-only) in DA neurons in the SNpc on the injected side (Fig. 8a). Western blots confirmed the expression of Flag-Syt11 3 weeks after virus injection, which did not affect the expression of endogenous Syt11 in this region (Fig. 8b). Interestingly, the conditional overexpression of Syt11 in SNpc DA neurons induced a dramatic decrease in ipsilateral striatal DA release (Fig. 8c) and an imbalance of contralateral motor stability (Fig. 8d–f), indicating that the accumulation of Syt11 in SNpc DA neurons is sufficient to mediate PD-related toxicity as well. Importantly, we also found that unilateral parkin KD in the SNpc (Fig. 8g) failed to impair the ipsilateral striatal DA release (Fig. 8h) and contralateral motor stability (Fig. 8i–m) in Syt11-cKO mice, demonstrating that Syt11 expression and accumulation in SNpc DA neurons is required for the pathogenesis caused by parkin deficiency.

## Discussion

Although loss-of-function mutations in *PARK2* are the most common causes of autosomal recessive PD^1,3,5,14,16^, the mechanisms underlying parkin-associated PD have puzzled the entire field for more than two decades. A major obstacle for mechanistic studies is that parkin-KO mice show minimal defects in striatal DA release and no loss of DA neurons in the SNpc, most probably due to compensation for parkin deficiency during development, because parkin KD (Fig. 7 and Supplementary Figs. 12 and 13) or conditional parkin-KO in the SNpc produced similar PD neurotoxicity in adult mice^17,20,40,41^. Here we produced a new adult mouse model for parkin-linked PD that is distinct from the traditional KO approaches and may represent the real conditions of PD with parkin mutations, which deserves a more thorough confirmation in future.

**Fig. 9:**
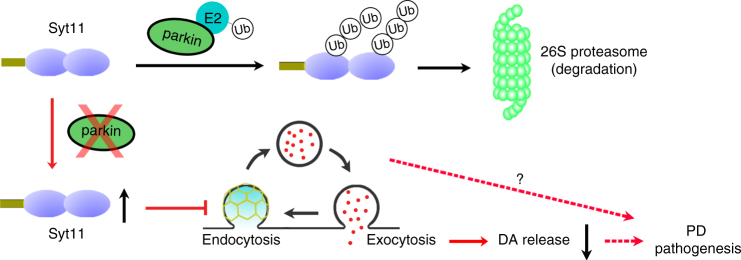
Schematic model showing the role of Syt11 in parkin-associated PD. The E3 ligase parkin mediates the ubiquitination and proteasome-dependent degradation of Syt11, whereas parkin dysfunction induced by genetic mutations or environmental factors leads to the accumulation of Syt11 in midbrain DA neurons. This inhibits endocytosis and the following processes of vesicle replenishment, which then lead to the impairment of DA release and most probably the initiation of PD pathogenesis

Many putative substrates of parkin have been reported^1–3,10,18,19^, but their pathogenic roles remain to be established due to scarce evidence in vivo. Our findings, for the first time, reveal that Syt11, a PD-risk factor based on meta-analysis^21^, is a physiopathological parkin substrate that mediates parkin-linked PD-like pathogenesis. Parkin dysfunction leads to the accumulation of Syt11, which inhibits endocytosis, vesicle replenishment, DA release from DA neurons, and finally initiates the pathogenesis of PD (Fig. 9). In addition to decreased DA transmission, the impaired endocytosis and vesicle recycling may also lead to a more general disturbance of the homeostasis of synaptic structure and membrane proteins^27^, thus whether the impaired DA release can fully explain the Syt11-mediated pathogenesis needs further investigation (Fig. 9). Meanwhile, other parkin substrates^1–3,10,18,20^ may also cooperate with Syt11 to mediate the pathogenesis of parkin-linked PD and thus contribute to parkin-linked neurotoxicity. Collectively, the present work not only validates Syt11 accumulation as a PD-risk factor but also uncovers a novel pathogenic pathway/mechanism for parkin-associated PD.

Many PD-risk genes and environmental factors have been identified, but it has been challenging to identify a central pathogenic pathway for PD. Our study reveals functional connectivity of the two PD-risk genes (*parkin* and *SYT11*) involving dysfunctional dopamine transmission, which is likely a common pathogenic pathway shared by other PD-risk genes (i.e. synaptojanin 1, *α-synuclein, TMEM230*, and *LRRK2*)^3,33,34,43–46^. Importantly, the progressive loss of DA neurons in the SNpc is markedly preceded by depression of DA transmission in Syt11-OE mice, indicating that the impaired endocytosis and vesicle recycling should be an early-stage event during PD pathogenesis. Our findings provided the first direct in vivo evidence that Syt11 mediates parkin-linked PD-like symptoms in mice and that the impaired vesicle recycling probably serves as a general early-stage pathogenic pathway for genetic causes of PD.

## Methods

### Plasmids

Myc-Syt11 was generated by PCR amplification of full-length rat synaptotagmin-11 (rSyt11, AF000423) from pCMV5-Sty11 and then ligated into pCMV5-Myc (Clontech). YFP-parkin, pcDH-ParkinR42P, and pcDH-ParkinR275W were kindly provided by Thomas L. Schwarz (Children’s Hospital Boston and Harvard Medical School)^10^ with the permission of Richard J. Youle (National Institutes of Health) and Matthew LaVoie (Brigham and Women’s Hospital and Harvard Medical School). Flag-Ub was kindly provided by Ruiping Xiao (Peking University). Synaptophysin-pHluorin was kindly gifted by Yongling Zhu (The Salk Institute for Biological Studies). For in vivo experiments, we used a lentiviral system, pFUGW, kindly provided by Chen Zhang (Peking University) with the permission of Thomas C. Sudhof (Stanford University), by substituting the EGFP coding sequence with that of Syt11-IRES2-EGFP or Syt11 (for Tf uptake). pFUGW itself served as control. A short-hairpin sequence targeting Syt11 (5′-GGCTGAGATCACAAATATACG-3′) was cloned into pMAGic 5.1 to silence Syt11 expression (shSyt11), and that targeting 5′-CCATCAAGAAGACCACCAA-3′ or 5′-CGATTCTGACACCAGCATCTT-3′ was cloned into pMAGic 4.1 for the KD of parkin (shParkin and parkin sh-2). Lentiviruses carrying shSyt11, shParkin, or shRNA control were produced by Shanghai Sunbio Biomedical Technology Co., Ltd (China). The C2B domain (288–430)-truncated form of Syt11^29^ was sub-cloned into the *Xho*I–*Mlu*I site of the vector pHBAAV-CAG-ZsGreen. The 3XFlag-tagged Syt11 fragment was sub-cloned into the *Kpn*I–*Mlu*I site of the vector pHBAAV-CAG-DIO-ZsGreen to generate plasmids overexpressing Cre-inducible Syt11. The AAV9 viral systems were from Hanbio Biotechnology Co., Ltd (China). All constructs were verified by DNA sequencing.

### Antibodies

The following primary antibodies were used: anti-β-actin (A5316; Sigma, 1:5000 dilution), anti-Syt11 (270,003; Synaptic System, 1:1000 dilution), anti-c-Myc (sc-789; Santa Cruz, 1:200 dilution, 1 μg mg^−1^ for IP), anti-c-Myc (M 4439; Sigma, 1 μg mg^−1^ for IP), anti-GFP (A11122; Invitrogen, 1:1000 dilution), anti-parkin (sc-32,282; Santa Cruz, 1:400 dilution), anti-Flag (F1804; Sigma, 1:1000 dilution), and anti-tyrosine hydroxylase (AB152; Millipore, 1:1000 dilution). IRDye 680CW goat anti-mouse IgG (LIC-926-32,220; LI-COR Biosciences, 1:5000 dilution) and IRDye 800CW goat anti-rabbit IgG (LIC-926-32,211; LI-COR Biosciences, 1:5000 dilution) were used for immunoblotting. Alexa Fluor® 488 goat anti-rabbit IgG (H+L) (A11034, 1:5000 dilution), Alexa Fluor® 594 goat anti-rabbit IgG (H+L) (A11037, 1:5000 dilution), and Alexa Fluor® 633 goat anti-rabbit IgG (H+L) (A-21,071, 1:5000 dilution) were used for immunostaining.

### Animals

The floxed Syt11-null mice were from The Jackson laboratory (strain B6.129-Syt11^tm1Sud^/J). TH-GFP and DAT-Cre transgenic mice were kindly provided by Dr. Minmin Luo (National Institute of Biological Sciences, China). Mice were housed in the animal facility with a maximum of five mice per cage under a 12-h light/dark cycle at 22 ± 2 °C. Food and water were available ad libitum. The use and care of animals was approved and directed by the Animal Care and Use Committee of Peking University and the Association for Assessment and Accreditation of Laboratory Animal Care.

### Cell culture and transfection

Human embryonic kidney 293A (HEK293A) and HEK293T cells were originally sourced from the ATCC and regularly checked for mycoplasma contamination. They were cultured in Dulbecco's modified Eagle's medium (DMEM) supplemented with 10% fetal bovine serum (FBS) at 37 °C in a humidified incubator (5% CO_2_). HEK293T cells were used for lentivirus production. For HEK293A cells, plasmids were transfected using VigoFect (Vigorous Biotechnology Beijing Co., China) when cells reached ~75% confluence following the manufacturer’s instructions. Immunoblotting and co-immunoprecipitation were performed 36 h after transfection.

Hippocampal neurons were prepared and cultured as described previously^29^. Briefly, hippocampi were dissected from postnatal day 0–1 Wistar rats and treated with 0.25% trypsin at 37 °C for ~12 min. Cells were plated on polyethyleneimine-coated glass coverslips and maintained in DMEM (Gibco) supplemented with 10% FBS for 3 h, which was then replaced by Neurobasal (Gibco) supplemented with 2% B27, 0.5 mM l-glutamine, and 5 μM cytosine arabinoside. Cultures at 5 days in vitro (DIV 5) were transfected with Lipofectamine 2000 (Invitrogen) according to the manufacturer’s instructions. TIRF and confocal imaging were performed at DIV 14.

Rat adrenal chromaffin cells (RACCs) were cultured as previously described with slight modifications^47^. Adult Wistar rats (female, ~250 g) were used and RACCs were isolated and digested in ice-cold D-Hanks containing collagenase type 1A (1 mg ml^−1^), DNase (10 mgml^−1^), and hyaluronidase (2 mg ml^−1^) at 37 °C for 40 min. Cells were dissociated, collected, and transfected with the Neon^TM^ 10 μl transfection system MPK1096 (Invitrogen). Cells were then plated on poly-l-lysine-coated coverslips and maintained in DMEM (Gibco) supplemented with 10% FBS (Gibco) and cysteine (6 mg l^−1^) at 37 °C in a humidified incubator (5% CO_2_).

### Lentivirus production and in vivo infection

Syt11-carrying and pFUGW control lentivirus were produced as previously described^48^. The lentiviral-expression vector and three helper plasmids (pRSV-REV, pMDLg-pRRE, and vesicular stomatitis virus G protein-expressing plasmid) were co-transfected into HEK293T cells at 4, 2, 2, and 2 μg of DNA per 25 cm^2^ culture area, using polyethylenimine “Max” (Mw 40,000, Cat 24,765-2; Polysciences) following the instructions of the manufacturer. Forty-eight hours after transfection, the culture medium was collected and clarified by centrifugation at 3000 *g* for 5 min. The supernatant was further centrifuged at 50,000 *g* for 120 min at 4 °C. The pellet was re-suspended in ice-cold phosphate-balanced saline (PBS, 1‰ volume of culture medium).

Stereotactic viral injections were carried out as previously described with small modifications^20^. Briefly, adult (~25 g) C57 male mice were anesthetized with urethane (1.5 g kg^−1^, i.p.) and body temperature was maintained at 37 °C using a heating pad (KEL-2000, Nanjing, China). Mice were fixed in a stereotaxic frame (Narishige, Japan) and leveled using the bregma and lambda landmarks. Craniotomies were performed to cause minimal damage to cortical tissue. A total of 2 μl viral suspension (titer: 10^8^–10^9^ per ml) was injected into the SNpc (3 mm posterior from bregma, 1.25 mm lateral, 4 mm ventral from the dura) at a rate of 100 nl min^−1^ using a 10 μl syringe with a blunt 32G needle. The needle was allowed to remain in place for another 20 min after injection, and then slowly withdrawn. Afterwards, the scalp was sutured and animals were placed on a 37 °C plate for recovery.

### Behavioral assays

All behavior experiments were performed in the afternoon. METH-induced asymmetric rotation tests^20,49^ were carried out 3, 4, 6, and 8 weeks after virus injection. The mice were placed in a quiet place for 30 min to habituate to the environment before intraperitoneal METH injection (8 mg kg^−1^). An unbiased observer counted the number of rotations for 90 min after injection. Rotation latency was recorded as the time from the METH injection to the appearance of the first asymmetric rotation and scored as 90 min if there was no rotation during the test.

Footprint gait was analyzed as previously described with slight modifications^33,34^. The hindpaws and forepaws were coated with black and red nontoxic paint. The mice were trained to walk along a 100-cm long and 10-cm wide open-top runway (with 10-cm high walls) with three runs per day for three consecutive days. A fresh sheet of white paper was placed on the floor of the runway for each run. The footprint patterns were assessed quantitatively by stride length and front/hind footprint overlap.

### Amperometric DA recording in striatal slices

Amperometric recordings in dorsal striatum slices were made using CFEs as described previously^32,35^. Mice were anesthetized with urethane (1.5 g kg^−1^, i.p.) and transcardially perfused with ~50 ml ice-cold artificial cerebrospinal fluid (“sectioning aCSF”) containing (in mM): 110 C_5_H_14_NClO, 2.5 KCl, 0.5 CaCl_2_, 7 MgCl_2_, 1.3 NaH_2_PO_4_, 25 NaCO_3_, 25 glucose (saturated with 95 O_2_ and 5% CO_2_). Then the brain was rapidly removed and cut into 300-μm horizontal slices on a vibratome (Leica VT 1000s; Nussloch, Germany). Slices containing the striatum were collected at +0.0 to +1.2 mm from bregma. Slices were allowed to recover for 30 min in “recording aCSF” (in mM): 125 NaCl, 2.5 KCl, 2 CaCl_2_, 1.3 MgCl_2_, 1.3 NaH_2_PO_4_, 25 NaCO_3_, 10 glucose (saturated with 95 O_2_ and 5% CO_2_) at 37 °C, and then kept at room temperature for recording. CFEs 7 μm in diameter with a ~200 μm sensor tip were used to measure DA release in the striatum. The exposed CFE tip was completely inserted into the subsurface of the striatal slice at an angle of ~30°. A holding potential of 780 mV was applied to the electrode by an EPC9/2 amplifier and controlled by Pulse software (HEKA Electronic, Lambrecht/Pfalz, Germany). Single electrical field stimulation (Estim) pulses (0.2 ms, 0.6 mA) or trains (10 pulses at 20 Hz) were delivered through a bipolar platinum electrode (150 μm in diameter) and generated by a Grass S88K stimulator (Astro-Med). The amperometric current (*I*_amp_) was low-pass filtered at 100 Hz and digitized at 3.13 kHz. The amplitude of amperometric current *I*_amp_ is proportional to the local DA overflow concentration [DA] with a calibration factor of 1 pA for ~6 nM. Off-line analysis was performed using Igor software (WaveMetrix).

### Amperometric recording of catecholamine release from RACCs

Standard polypropylene-insulated CFEs (5 μm in diameter, Dagan Instruments, Minneapolis, USA) were used to record the quantal release of catecholamines from single RACCs as we described previously^47^. The CFE sensor tip was lightly positioned on the surface of an RACC. The electrode was held at 780 mV. The output *I*_amp_ signal was low-pass filtered at 300 Hz and digitized at 2 kHz. For statistical analysis of the kinetic properties of amperometric spikes, only events >10 pA were used.

### Immunohistochemistry

Immunohistochemistry was performed as described previously with slight modifications^32–34^. Briefly, mice were anesthetized and perfused with 0.9% saline followed by 4% paraformaldehyde in PBS, and the brain was removed and post-fixed overnight in 4% paraformaldehyde in PBS at 4 °C. After dehydration in 10, 20, and 30% sucrose, a series of coronal sections (40 μm thick) across the striatum and midbrain were cut on a Leica cryostat (every fourth section was used for counting dopaminergic neurons). The sections were permeabilized with 0.3% Triton X-100 in PBS containing 2% bovine serum albumin (BSA) for 5 min at room temperature. After blocking with 2% BSA in PBS, the samples were incubated with primary antibodies at 4 °C overnight. After three washes with blocking solution, samples were incubated for 1 h with secondary antibodies. The sections were subjected to TUNEL staining (Cat. No. 12 156 792 910; Roche) following the manufacturer’s protocols. Nuclei were visualized by DAPI staining and samples were mounted on slides with 50% glycerol. Fluorescence images were captured using a Zeiss 710 inverted confocal microscope. All paired images were captured at the same gain and offset settings and post-collection processing was applied uniformly to all paired images using ImageJ (National Institutes of Health, Bethesda, MD).

### Transferrin uptake

The transferrin (Tf) uptake essay was modified from that previously described^50^. Syt11-carrying lentivirus was unilaterally injected into the SNpc of TH-GFP mice (provided by Minmin Luo, National Institute of Biological Sciences, China). The SNpc-containing slices were placed on Millicell^®^ CM (0.4 μm pore size; Millipore) culture plates and incubated with DMEM supplemented with 10% FBS at 37 °C in a humidified incubator (5% CO_2_). After 4-h incubation, the slices were serum-starved by replacing the medium with serum-free DMEM containing 20 mM HEPES and 1 mg ml^−1^ BSA for 45 min at 37 °C. They were then incubated with serum-free medium containing 25 μg ml^−1^ human Tf conjugated to Alexa Fluor 594 (Invitrogen) for 30 min at 37 °C. The unbound Tf was washed off with ice-cold PBS containing 0.3 mM CaCl_2_ and 0.3 mM MgCl_2_. The slices were subsequently fixed in an ice-cold solution of 4% formaldehyde and mounted on slides with 50% glycerol. A ×42 oil lens on a Zeiss 710 inverted confocal microscope was used to scan z-stack of 1-μm optical sections. Identical settings were applied to all samples in each experiment. The average fluorescence intensity of the Tf signal in GFP-positive cell bodies in the SNpc was calculated with ImageJ.

### TIRF imaging

Hippocampal neurons transfected with plasmid expressing Clathrin-DsRed with or without the co-transfection of plasmid expressing Syt11 were cultured for 12–18 h. TIRF imaging was performed on an inverted microscope with a ×100 TIRF objective lens (Olympus IX-81; numerical aperture 1.45) as described^51,52^. Images were captured by an Andor EMCCD using Andor iQ software with an exposure time of ~100 ms. The temperature of the cell under imaging was kept at ~35 °C by a laboratory-made heater attached on the objective lens. Standard or 70 mM KCl-containing external solution (in mM, 85 NaCl, 70 KCl, 2.5 CaCl_2_, 1 MgCl_2_, 10 H-HEPES, and 10 d-glucose, pH 7.4) was applied using a gravity-fed perfusion system (MPS-2, Yibo Inc., Wuhan, China). For the analysis of single endocytic events, each event was selected and marked with 2.8-μm-diameter circular area. Fluorescence intensity values were calculated and analyzed using ImageJ. The whole-cell endocytosis was calculated based on the fluorescence changes of clathrin-DsRed on the entire cell surface.

### Confocal live imaging

Hippocampal neurons were continuously perfused with standards bath solution containing: 140 mM NaCl, 5 mM KCl, 2 mM CaCl_2_, 2 mM MgCl_2_, 10 mM H-HEPES, 10 mM d-glucose, 10 μM CNQX, and 50 μM D-AP5, pH 7.4. Neurons were stimulated with 100 pulses delivered at 20 Hz (100 mA, 1-ms pulse width) via two parallel platinum wires embedded in the imaging chamber. Time-lapse images were captured at 2-s intervals through the ×42 oil-immersion lens of the Zeiss 710 inverted confocal microscope. Fluorescence changes at individual boutons were monitored over time and calculated as normalized Δ*F*. To establish a stable baseline, images were acquired for 20 s prior to stimulation. After collection, data were analyzed in ImageJ and Adobe Photoshop.

### Protein preparation and western blotting

Cultured cells were washed with PBS and homogenized on ice with lysate buffer (20 mM HEPES at pH 7.4, 100 mM KCl, 2 mM EDTA, 1% NP40, 1 mM PMSF, and 2% proteinase inhibitor (539,134; Calbiochem)). Mice were anesthetized and perfused with ~50 ml ice-cold sectioning aCSF. Then the brain was rapidly removed and cut into 200-μm horizontal slices on a vibratome (Leica VT 1000s, Nussloch, Germany). Slices containing the midbrain were collected. For parkin KD and parkin/Syt11 DKD mice, the contralateral and ipsilateral SNpc were separately collected under an Olympus IX-70 inverted microscope for homogenization. The homogenates were centrifuged at 13,500 rpm for 15 min at 4 °C and the supernatants were collected and boiled in SDS-PAGE buffer. Proteins were electrophoresed and transferred to nitrocellulose filter membranes. The membranes were blocked by incubation for 1 h with PBS containing 0.1% Tween-20 (v/v) and 5% non-fat dried milk (w/v), then incubated with primary antibodies followed by secondary antibodies. Blots were scanned with an Odyssey infrared imaging system (LI-COR Biosciences) and quantified with ImageJ. Images have been cropped for presentation. Full size images are presented in Supplementary Fig. 14.

### Co-immunoprecipitation and ubiquitination assay

HEK293A cells transfected with the indicated plasmid combinations were treated with 10 μM MG132 for 12 h before lysis. Cells were homogenized on ice with lysate buffer, followed by centrifugation at 13,500 rpm for 15 min. The supernatant was pre-cleaned with Protein G/A Sepharose 4 Fast Flow (17-6002-35; GE Life Sciences) for 1 h at 4 °C, and incubated with the indicated primary antibodies and Protein G/A agarose beads for 4 h at 4 °C. The resulting immunoprecipitates were washed three times with lysate buffer and protein was eluted by adding sample loading buffer and heating at 100 °C for 6 min. The samples were then electrophoresed and transferred to nitrocellulose filter membranes for western blot analysis.

### Statistics

All experiments were performed with side-by-side controls and in random order, and were replicated at least three times. Sample sizes are consistent with those reported in similar studies. No samples or animals that provided successful measurements were excluded from analysis. Experiments did not involve blinding because we used GFP as a marker for in vivo genetic modulation (unilateral virus injection) and no protein expression, neuron numbers, or behavioral performances were predefined. Statistical comparisons were performed with the two-tailed paired/unpaired Student’s *t*-test, the Wilcoxon–Mann-Whitney nonparametric test, the Kolmogorov–Smirnov test, or one-way ANOVA (followed by the LSD test for between-group comparisons) as indicated. Shapiro–Wilk test was used to test normality of data, and Lenene’s test to assess the equality of variance. All tests were performed using SPSS (Statistical Package for the Social Sciences) 13.0. Significant differences were accepted at *P* < 0.05. Data are shown as mean ± s.e.m. Numbers of mice or cells analyzed are indicated in the figures and/or legends.

### Data availability

The authors declare that all the data supporting the findings of this study are available within the article and its Supplementary Information files and from the corresponding author on reasonable request.

## Electronic supplementary material

Supplementary Information
