# Supplementary material for: Synaptotagmin-11 is a critical mediator of parkin-linked neurotoxicity and Parkinson’s disease-like pathology
Source: Nat Commun. 2018 Jan 8;9:81. doi: 10.1038/s41467-017-02593-y (PMC5758517; doi:10.1038/s41467-017-02593-y)
Supplement: Supplementary file 1 — Supplementary Information [file 41467_2017_2593_MOESM1_ESM.pdf]

SUPPLEMENTARY INFORMATION

Supplementary Figure 1

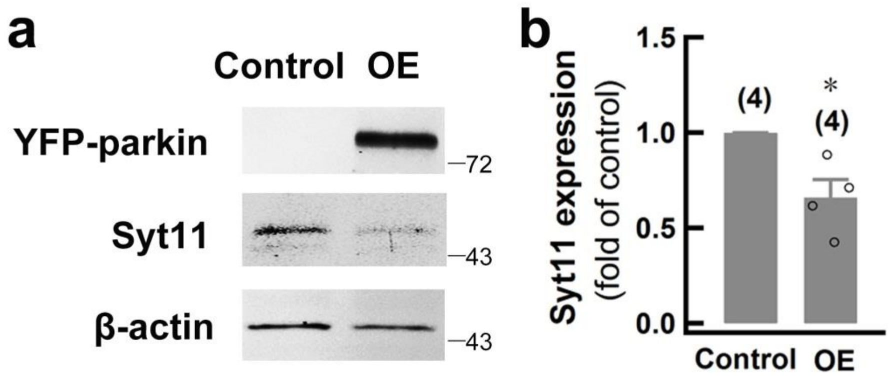

Supplementary Figure 1. Parkin over-expression decreased the expression of endogenous Syt11 in hippocampal neurons. (**a**) Representative western blots showing the expression of Syt11 in parkin over-expressing hippocampal neurons. (**b**) Statistics showing the expression of Syt11 as in (**a**). Data are shown as mean ± s.e.m. Student's *t*-test, *P <0.05.

Supplementary Figure 2

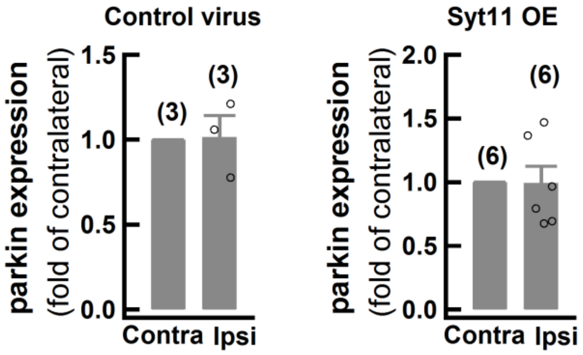

Supplementary Figure 2. Statistics showing the expression of parkin in the bilateral SNpc from mice unilaterally overexpressing (OE) Syt11 and controls. Data are shown as mean ± s.e.m. Paired Student's *t*-test, P = 0.918 for control virus, P = 0.963 for Syt11 OE.

Supplementary Figure 3

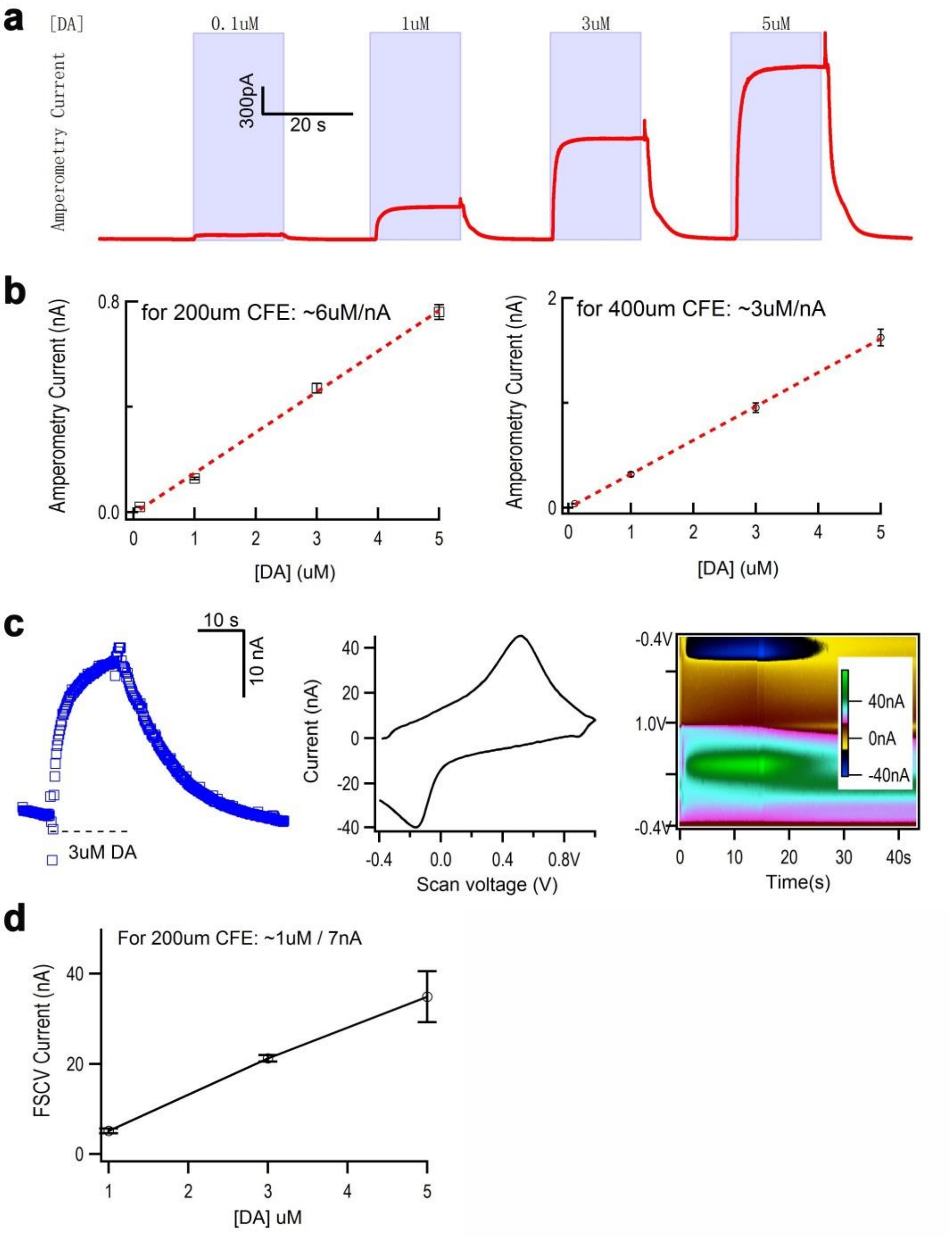

Supplementary Figure 3. DA calibration by amperometry and fast scan cyclic voltammetry (FSCV) *in vitro*. (**a**) Typical trace of amperometric recordings of DA. CFE length ~400 µm. (**b**) Statistics of DA calibration *in vitro* with CFE amperometric recordings (left, 6 µM/nA with ~200 µm CFE; right, 3 µM/nA with ~400 µm CFE). (**c**) Representative FSCV recording of 3 µM DA *in vitro*. Left, I-t curve of 3 µM DA; middle, I-V curve of 3 µM DA; right, voltage scan color image of FSCV recording. CFE length ~200 µm. (**d**) Statistics of *in vitro* DA calibration by CFE FSCV recordings (~0.15 µM/nA) as in (**c**). Data are shown as mean ± s.e.m.

Supplementary Figure 4

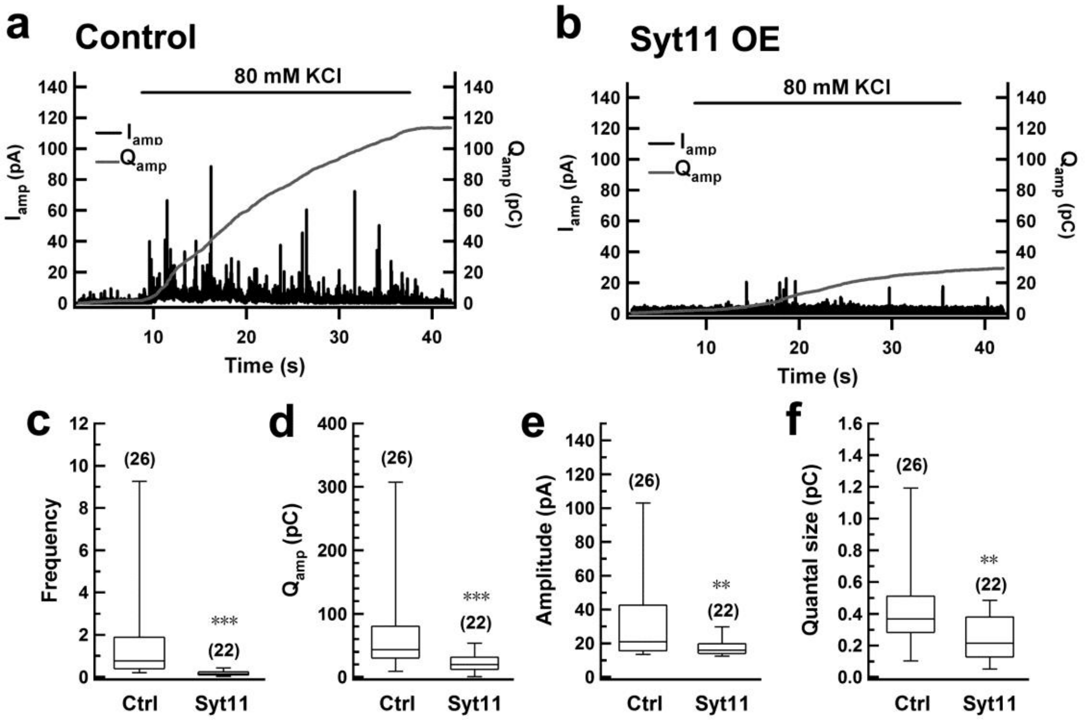

Supplementary Figure 4. Syt11 over-expression inhibits catecholamine secretion in adrenal chromaffin cells. (**a**,**b**) Representative and cumulative amperometric current traces of catecholamine release from control (n = 26) and Syt11 over-expressing (OE, n = 22) rat chromaffin cells. (**c**-**f**) Statistics of release frequency (P <0.001), total secretion (P <0.001), single vesicle quantal amplitude (P = 0.006), and quantal size (charge, P = 0.003) recorded as in (**a**) and (**b**). Mann-Whitney test, box and whisker plots show medians (central line in the box), ranges between 25th and 75th percentiles (box) and minimum–maximum ranges (whiskers), **P <0.01, ***P <0.001.

Supplementary Figure 5

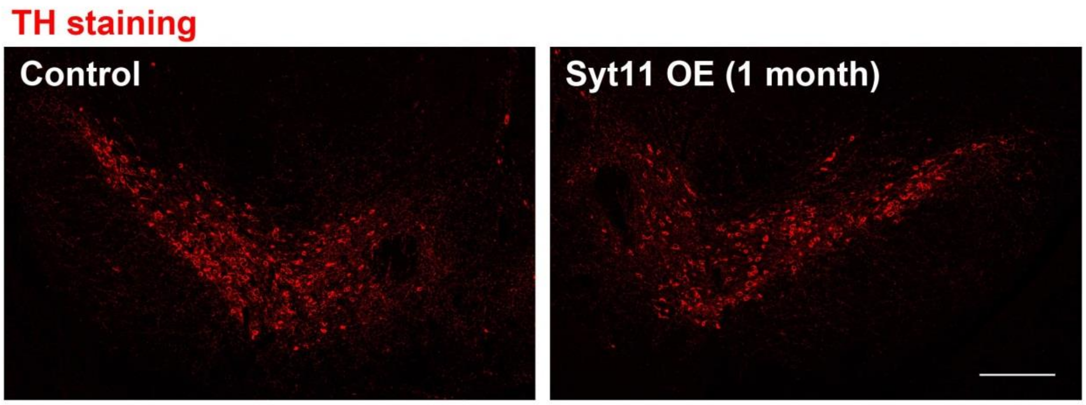

Supplementary Figure 5. Representative micrographs showing normal TH staining in the Syt11-overexpressing SNpc 1 month after virus injection. Syt11-carrying lentivirus was unilaterally injected into the SNpc and TH staining was performed on SNpc-containing horizontal slices 1 month after virus injection. Scale bar, 200 µm.

Supplementary Figure 6

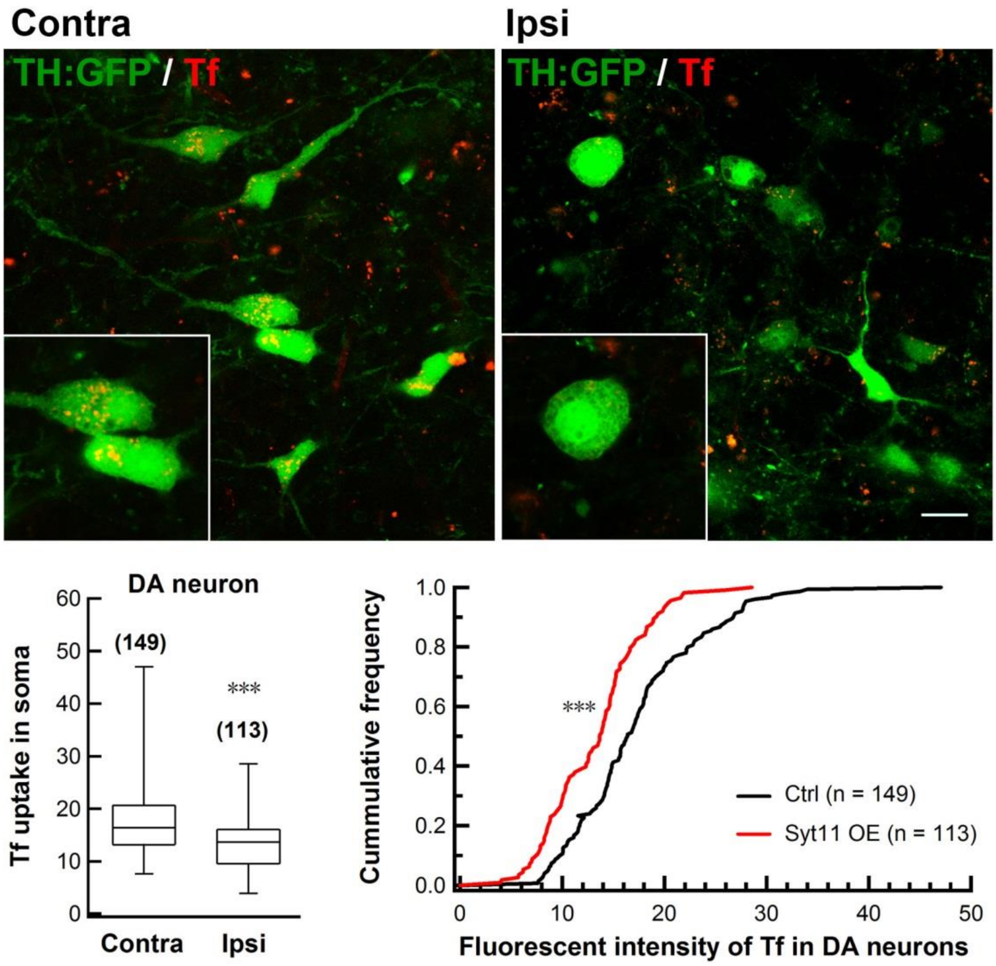

Supplementary Figure 6. Syt11 inhibits clathrin-mediated endocytosis of DA neurons in the SNpc. Transferrin (Tf) uptake of DA neurons in the Syt11-overexpressing or contralateral SNpc in TH-driven GFP transgenic (TH-GFP) mice. Syt11-carrying lentivirus was unilaterally injected into the SNpc in TH-GFP mice and Tf uptake was assessed in SNpc-containing horizontal slices 1 month later. Scale bar, 20 μm. n represents number of neurons. Mann-Whitney test (lower left panel) and two-sample Kolmogorov-Smirnov test (lower right panel), ****P* <0.001.

Supplementary Figure 7

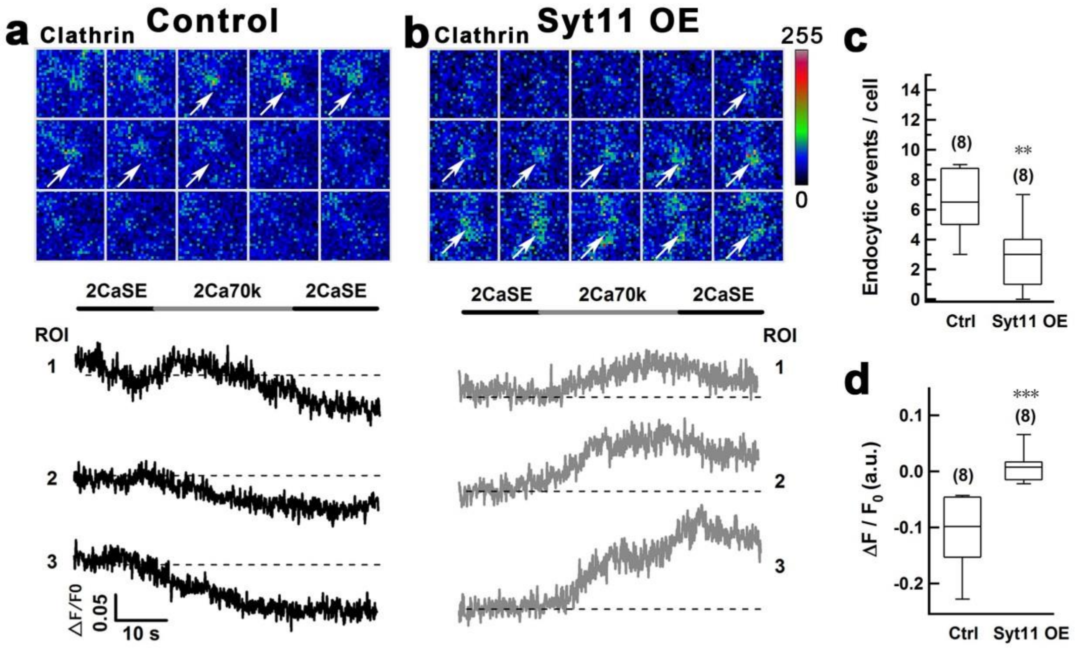

Supplementary Figure 7. Syt11 inhibits clathrin-mediated endocytosis in hippocampal neurons. (**a**,**b**) TIRF imaging of clathrin-DsRed in soma of hippocampal neurons with or without Syt11 overexpression (OE). Representative pseudocolor TIRF image frames (2.8 µm × 2.8 µm, 10 s/frame) and fluorescence traces of clathrin-DsRed clusters in somata of hippocampal neurons (control, Ctrl) or that overexpressing Syt11 (Syt11 OE). Arrows indicate assembled clathrin clusters, which were endocytosed into the cytosol in control cells (**a**) but clamped on the plasma membrane in Syt11-OE cells (**b**) in response to 70 mM-KCl (2Ca70K) stimulation. The fluorescence failed to decay back to baseline in Syt11-OE cells. (**c**) Statistics of endocytic events/cell in control and Syt11-OE neurons. Endocytic events were identified as those with fluorescence decay back to or below baseline. (**d**) Statistics of $\Delta F/F_0$ in control and Syt11-OE neurons, in which ΔF was calculated as the fluorescence change of clathrin-DsRed on the entire cell surface before and 25 s after KCl stimulation, and F_0 was the value before KCl stimulation. Mann-Whitney test, box and whisker plots show medians (central line in the box), ranges between 25th and 75th percentiles (box) and minimum–maximum ranges (whiskers). $^{**}P < 0.01$, $^{***}P < 0.001$.

Supplementary Figure 8

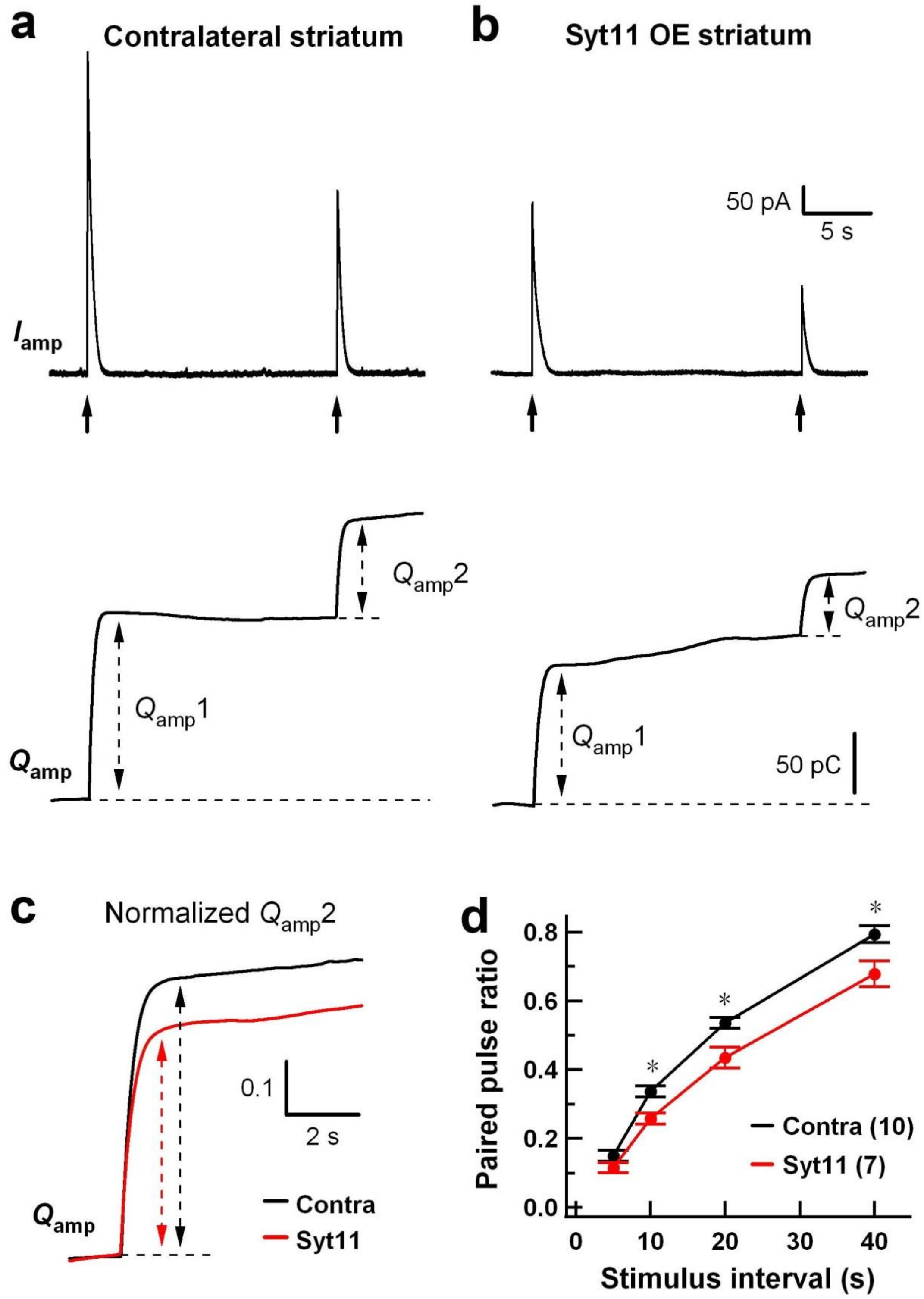

Supplementary Figure 8. Syt11 inhibits vesicle recycling in striatal DA terminals. (**a**,**b**) Paired-pulse stimulus (arrows)-induced amperometric currents (I_{amp}, upper panels) and the cumulative traces (Q_{amp}, lower panels) recorded contralateral and ipsilateral to the SNpc with Syt11-overexpression. (**c**) The normalized $Q_{amp}2$ (total DA release in response to the 2nd stimulus) normalized by $Q_{amp}1$ (total DA release in

response to the 1st stimulus) was used to assess the reduced paired-pulse ratio by Syt11 OE. (**d**) Paired-pulse ratio of DA release with different inter-stimulus intervals (P = 0.075, 0.021, 0.016, and 0.022 for 5, 10, 20, and 40 s). Data were collected from 4 Syt11- OE mice, and n represents number of brain slices. Data are shown as mean ± s.e.m. Unpaired Student's *t*-test, *P <0.05.

Supplementary Figure 9

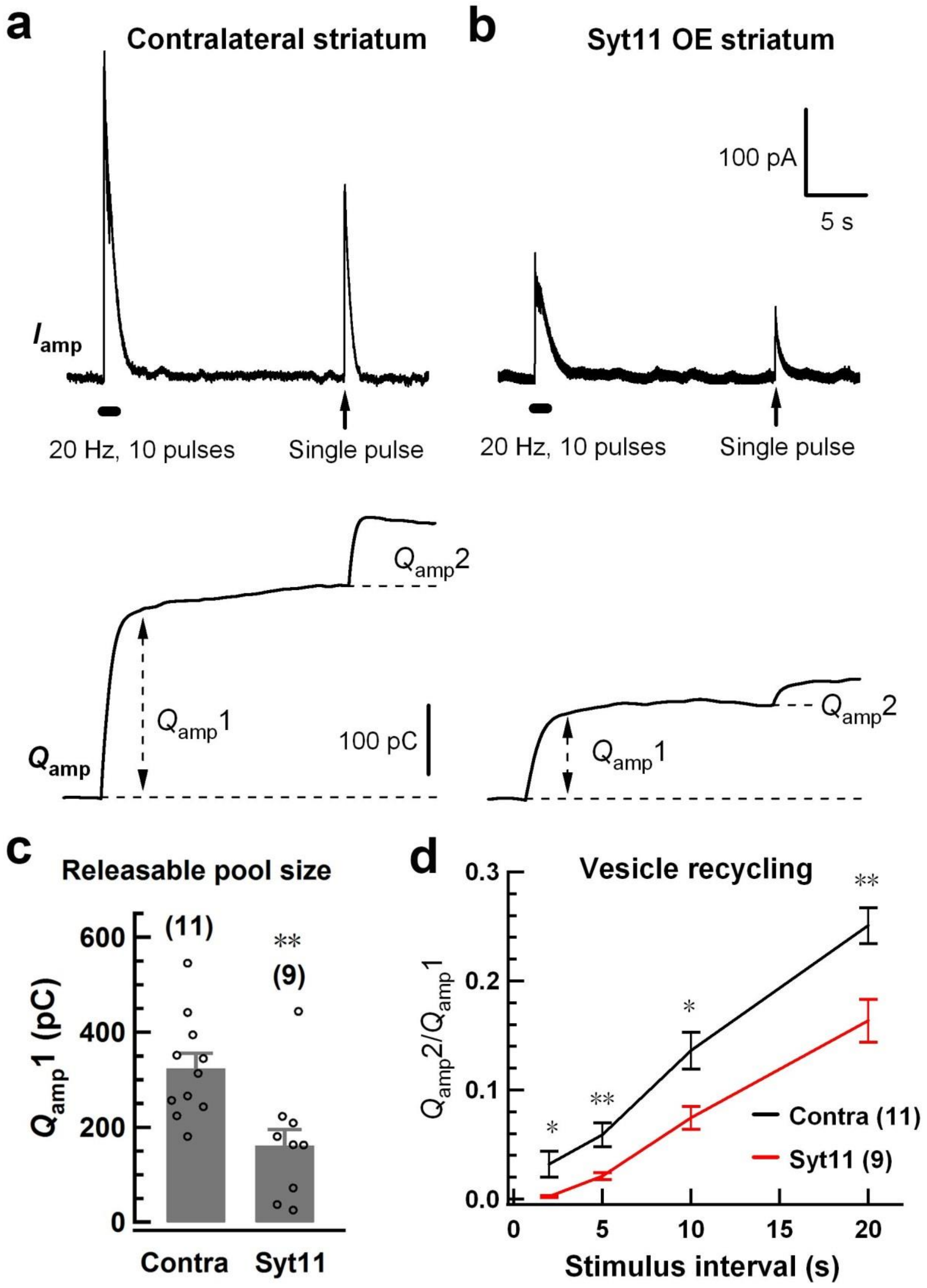

Supplementary Figure 9. Syt11 overexpression decreases the vesicle pool size of striatal dopaminergic terminals. (**a**,**b**) A burst of pulses (20 Hz, 10 pulses) followed by single-pulse stimulation evoked DA release (upper panels, I_{amp}; lower panels, Q_{amp}) recorded in the striatum contralateral (**a**) or ipsilateral (**b**) to the SNpc with Syt11-overexpression (OE). (**c**) Releasable vesicle pool size ($Q_{amp}1$) reduced by

Syt11-OE (P = 0.003). (**d**) Recovery rate of readily-releasable vesicle pools ($Q_{amp}2/Q_{amp}1$, ratio of DA release to vesicle pool size) at different interstimulus intervals (P = 0.026, 0.007, 0.012 and 0.007 for 2, 5, 10 and 20 s). Data were collected from 3 Syt11-OE mice, and n represents number of brain slices. Data are shown as mean ± s.e.m. Unpaired Student's *t*-test, $^{*}P$ <0.05, $^{**}P$ <0.01.

Supplementary Figure 10

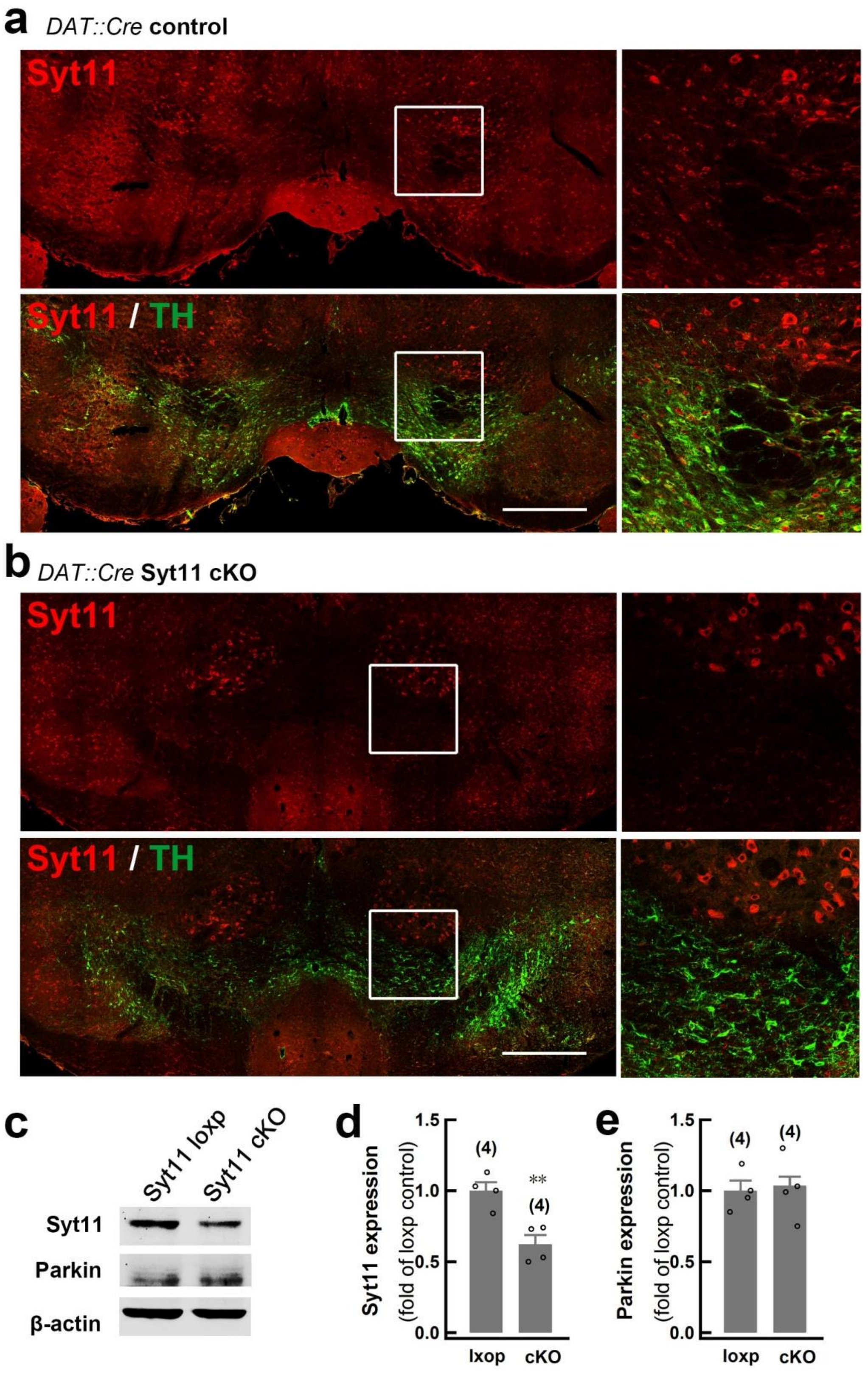

Supplementary Figure 10. Characterization of DAT-driven Syt11 conditional KO mice. (**a**,**b**) Representative micrographs showing the specific absence of Syt11 staining in TH-positive neurons in the SNpc from Syt11 cKO mice. Homozygous

floxed Syt11-null mice were crossed with DA transporter-driven Cre heterozygous knockin (DAT-Cre) mice to produce DA neuron-restricted Syt11-conditional knockout mice (Syt11 cKO). Scale bars, 500 μm. (**c-e**) Representative western blots and statistics showing the expression of Syt11 (P = 0.005) and parkin (P = 0.818) in the bilateral SNpc from Syt11 cKO mice. n represents number of mice (biological repeats). Data are shown as mean ± s.e.m. Unpaired Student's *t*-test, $^{**}P$ <0.01.

Supplementary Figure 11

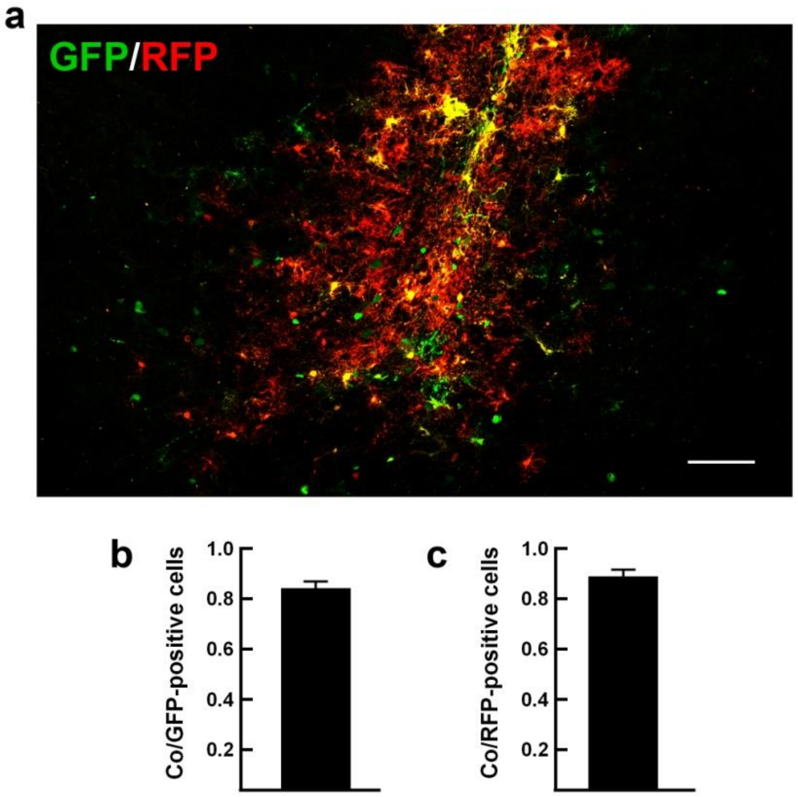

Supplementary Figure 11. Co-infection efficiency of two lentiviruses *in vivo*. (**a**) Representative micrograph of an SNpc-containing slice showing the infection of GFP-expressing and RFP-expressing scrambled shRNA control lentiviruses in the SNpc *in vivo*. Scale bar, 500 μm. (**b**) Statistics of the ratio of co-infected neurons to that of GFP-positive or RFP-positive neurons. Data are shown as mean ± s.e.m.

Supplementary Figure 12

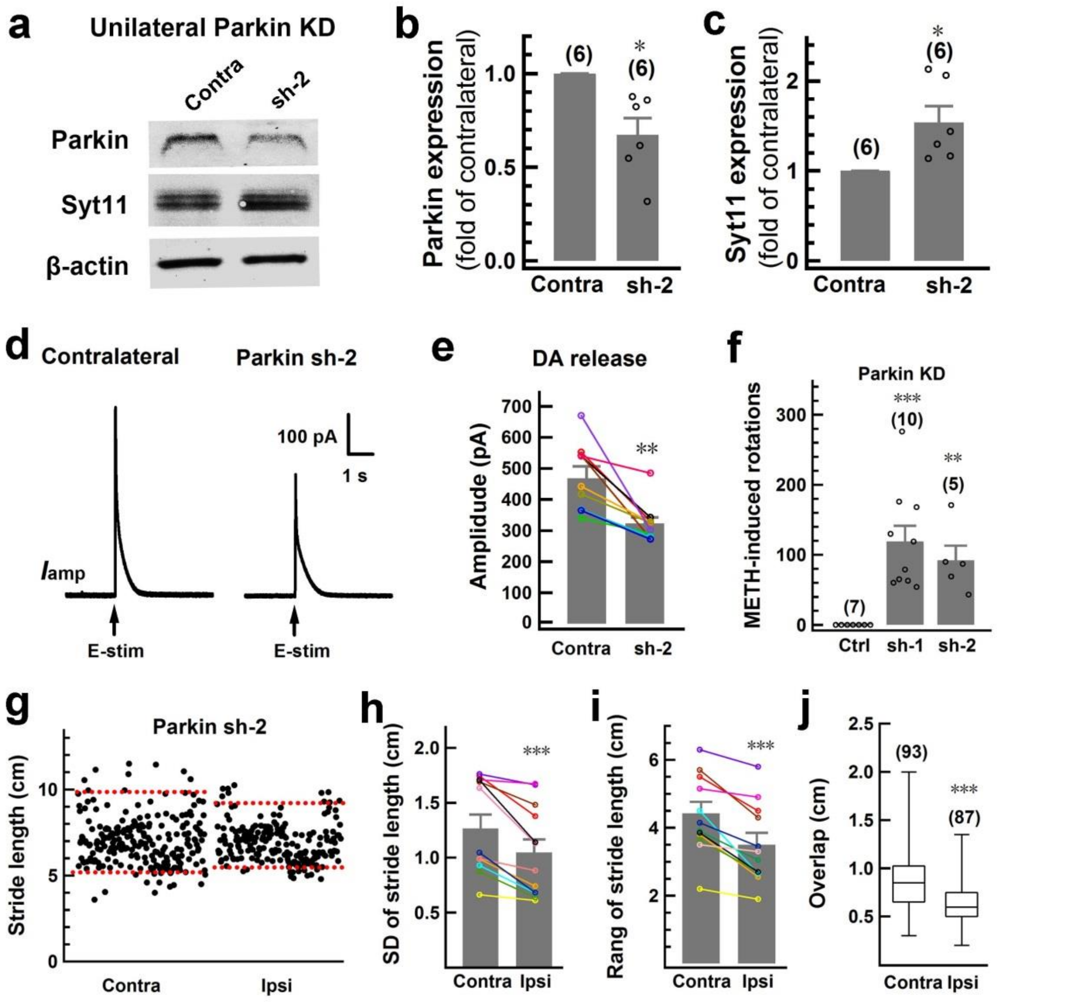

Supplementary Figure 12. Parkin KD induces pathogenesis of PD. (**a-c**) Representative western blots and statistics showing the expression of parkin (P = 0.015) and Syt11 (P = 0.032) in the bilateral SNpc from parkin KD mice with an independent parkin-targeting shRNA (parkin sh-2). Western blots were performed 1 month after virus injection. (**d**,**e**) Representative amperometric currents (I_{amp}) and statistics showing the impaired DA release from parkin KD dopaminergic terminals (with parkin sh-2) in the striatum (P = 0.004). (**f**) METH-induced asymmetric rotation (in 90 min) of parkin KD mice. A scrambled non-targeting shRNA served as negative control (Ctrl) for parkin KD (P = 0.001). (**g-j**) Footprint data showing that unilateral parkin KD in the SNpc with sh-2 induced motor defect in the contralateral limbs(P <0.001). Dashed red lines show the 5% and 95% percentiles of stride length in (**g**). n = 93 Contra and 87 Ipsi steps from 6 mice in (**j**). Data are shown as mean ± s.e.m. for (**b-i**). Paired Student's *t*-test for (**b**,**c**,**e**,**h**,**i**); one-way ANOVA for (**f**); Mann-Whitney test for (**j**), box and whisker plots show medians (central line in the box), ranges between 25th and 75th percentiles (box) and minimum–maximum ranges (whiskers). *P <0.05, **P <0.01, ***P <0.001.

Supplementary Figure 13

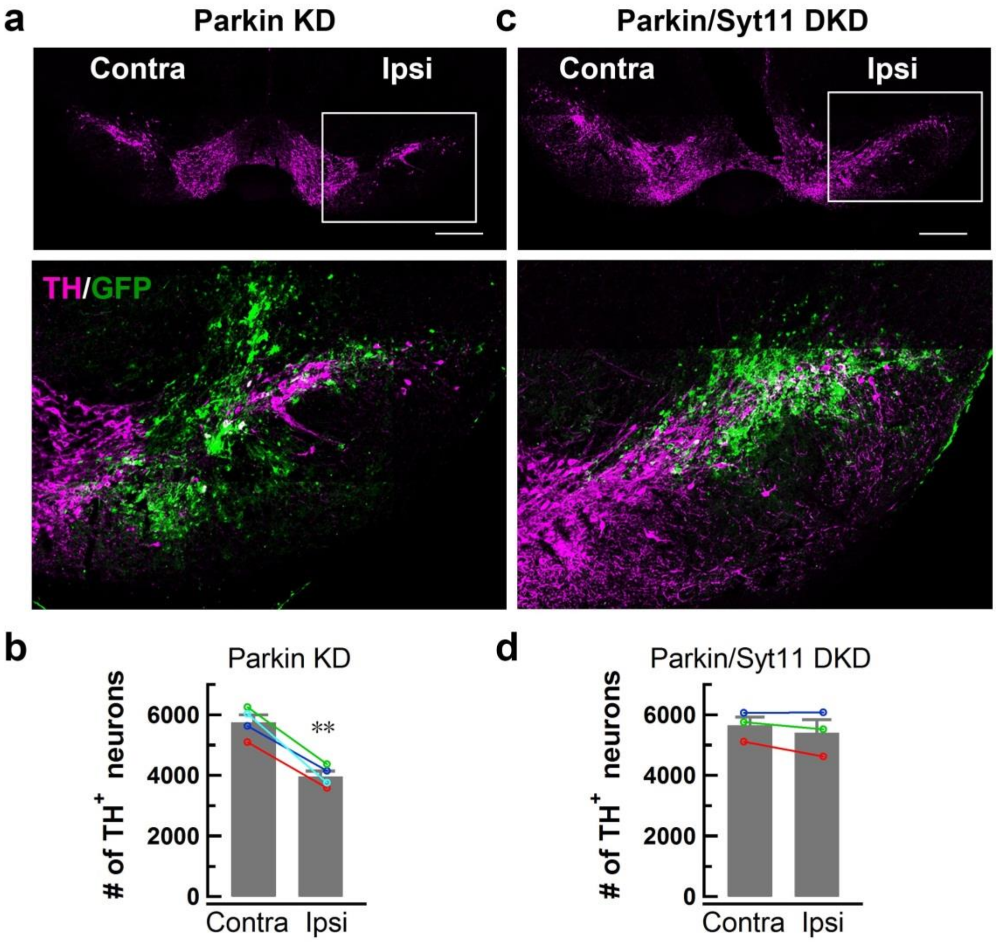

Supplementary Figure 13. Syt11 KD rescues parkin KD-induced DA neuron loss. (**a**,**b**) Lentiviruses carrying shParkin and those carrying control shRNA were mixed in a 1:1 ratio and unilaterally injected into the SNpc. DA neurons with TH staining were counted on both sides 3 months later (P = 0.002). GFP was used to indicate infected neurons. (**c**,**d**) Similar to (**a**,**b**), except that shParkin- and shSyt11-carrying lentiviruses were used (P = 0.250). Scale bars, 500 µm. Data are shown as mean ± s.e.m. Paired Student's *t*-test, *P <0.05.

Supplementary Figure 14

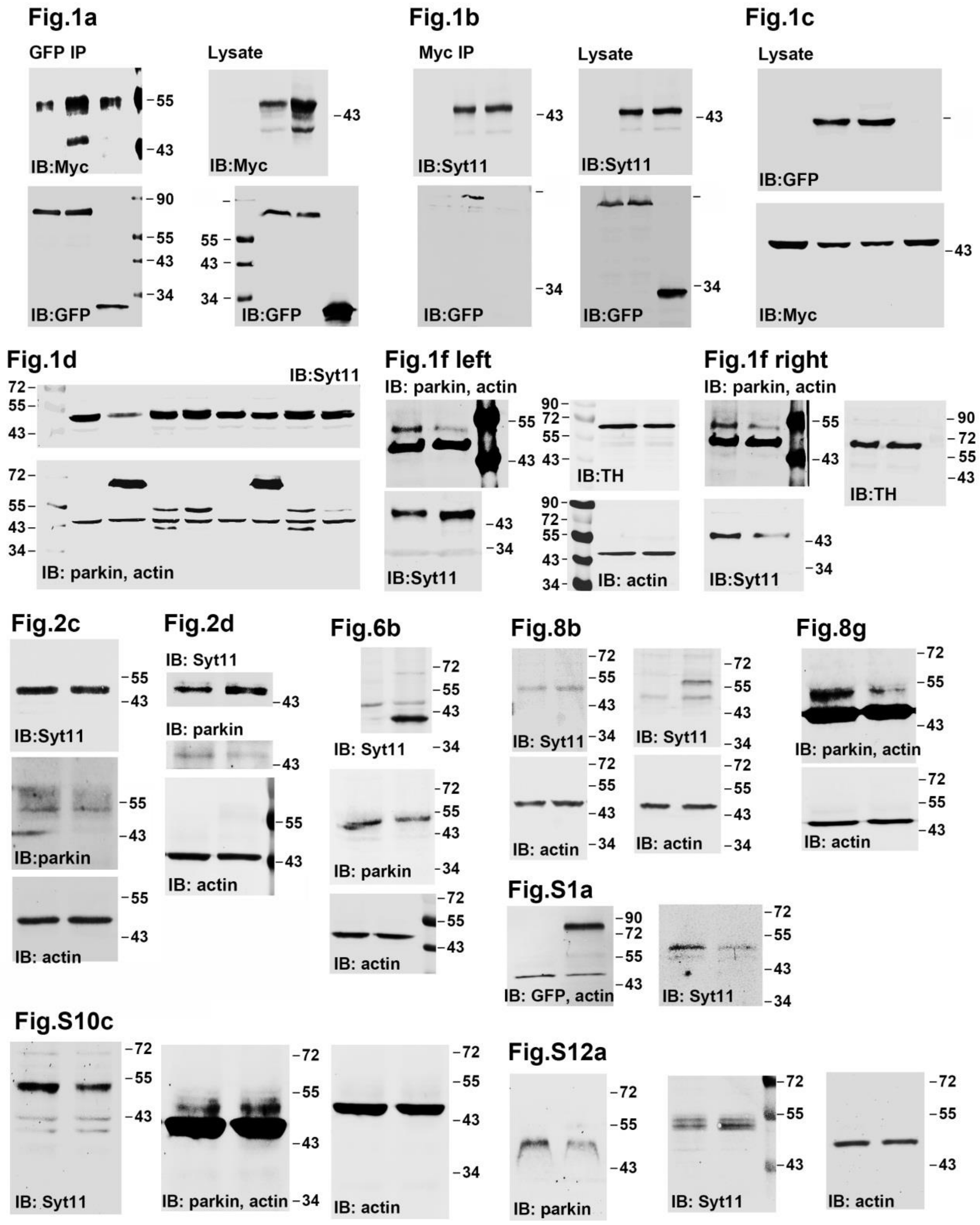

Supplementary Figure 14. Uncropped scans of the most important blots.
